# Effects of Metabolic Pathways and Products on Early Mammalian Embryonic Development

**DOI:** 10.3390/cells15191808

**Published:** 2026-10-03

**Authors:** Hu Tao, Zaidong Hua, Jingwei Wei, Xiangxing Zhu, Qi Xiong

**Affiliations:** 1 Key Laboratory of Animal Embryo Engineering and Molecular Breeding of Hubei Province, Institute of Animal Sciences and Veterinary Medicine, Hubei Academy of Agricultural Sciences, Wuhan 430064, China; taohu@hbaas.com (H.T.);; 2 Animal Science Institute, Guangxi University, Nanning 530005, China; 3 Guangdong Provincial Engineering and Technology Research Center for Gene Editing, School of Medical Engineering, Foshan University, Foshan 528225, China

**Keywords:** embryonic development, metabolism, cell lineage, embryology

## Abstract

Background: The study of early mammalian embryonic development is the basis for the cultivation of embryonic stem cells (ESCs), assisted reproductive technology (ART), and understanding and preventing early embryonic developmental defects. Metabolism is the most fundamental physiological activity of embryonic development. Studies have shown that specific metabolic pathways and metabolites play key roles in embryonic development. These roles involve the regulation of signal transduction in addition to the provision of energy to the organism. Aim of Review: This review examines the factors affecting the critical moments of embryonic development, such as oocyte maturation, zygotic gene activation, cell compaction, and two genealogical differentiations. It also presents the characteristics of embryonic metabolism at this time. This review aims to provide new insights and approaches for improving early mammalian embryonic development by clarifying the relationship between embryonic metabolism and its developmental processes. Furthermore, the review emphasizes embryonic metabolism as a potential indicator for assessing the level of embryonic development in vitro, providing a new basis for assessment in assisted reproductive technologies. Key Scientific Concepts of Review: This review is focused on three main key concepts. First, we demonstrate known regulatory mechanisms of embryonic development to enhance the potential of early embryonic development. Second, we elucidate the potential role of metabolic processes and their products in early embryonic development. Third, we provide new foundations and perspectives for the study of the early embryo from the perspective of cellular metabolism.

## 1. Introduction

Early mammalian embryonic development refers to a series of developmental events from the union of two terminally differentiated gametes forming a zygote to embryo implantation. Many factors with many unknown mechanisms regulate this process.

### 1.1. Process of Embryo Development

#### 1.1.1. Folliculogenesis

**Figure 1:**
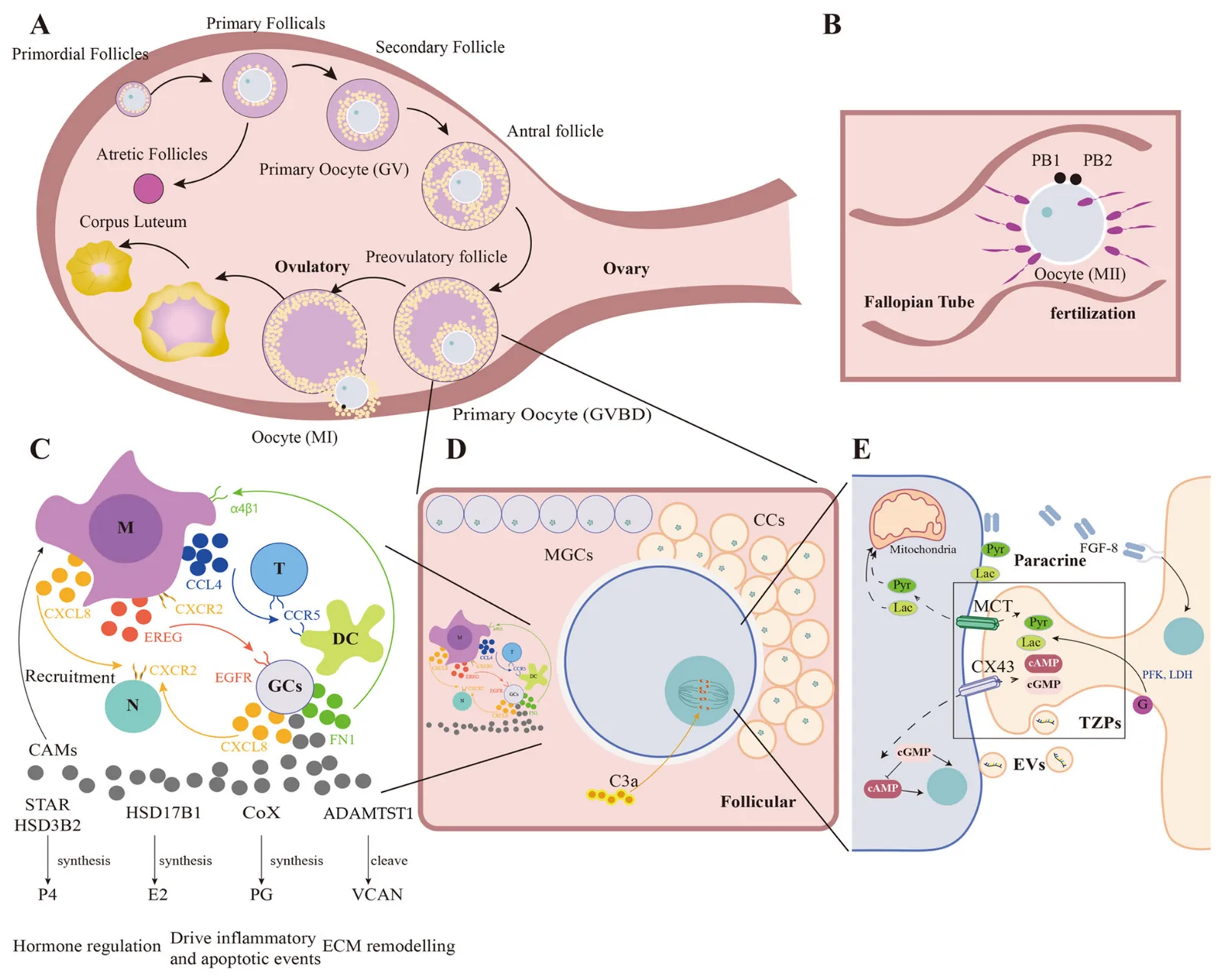
The process of oocyte maturation in mammals. (**A**) Follicular development. (**B**) Fertilization. PB1: First polar body; PB2: second polar body. (**C**): Function of GCs and cells in follicular fluid. GCs: Granulosa cells; N: neutrophil; M: macrophage; T: T cells; DC: dendritic cells; CAMs: cell adhesion molecules; STAR: steroidogenic acute regulatory protein; HSD3B2: hydroxy-delta-5-steroid dehydrogenase; HSD17B1: hydroxysteroid 17-beta dehydrogenase 1; CoX: cyclooxygenase; ADAMTST1: ADAM metallopeptidase with thrombospondin type 1 motif 1; FN1: fibronectin 1; α4β1: FN1-α4β1 complex; CCL4: C-C motif chemokine ligand 4; CCR5: C-C motif chemokine receptor 5; CXCR2: C-X-C motif chemokine receptor 2; CXCL8: C-X-C motif chemokine ligand 8; EREG: epiregulin; EGFR: epidermal growth factor receptor; VCAN: Versican; PG: prostaglandin; ECM: extracellular matrix; E2: estrogen; P4: progesterone. (**D**) Preovulatory follicle. CCs: Cumulus cells; MGCs: mural granulosa cells; C3a: C3a-peptide. (**E**) Communication pathways between oocyte and GCs. TZPs: Transzonal projections; EVs: extracellular vesicle; G: glucose; PFK: phosphofructokinase; LDH: lactate dehydrogenase; Pyr; pyruvic acid; Lac: lactic acid; MCT: monocarboxylate transporter; CX43: connexin 43; cAMP: cyclic adenosine monophosphate; cGMP: cyclic guanosine monophosphate.

In female viviparous mammals, germ cells grow and develop in the ovary, where the oogonium and monolayer of squamous granulosa cells (GCs) are assembled and maintained as the primordial follicle after birth, usually within 2–3 days after mouse birth [1], at 17–20 weeks of human fetal life [2] and 56 to 70 days of pig life [3], and are confined to the basement membrane [4]. Primordial follicles gradually develop into primary follicles, with the cuboidization of peripheral squamous GCs [5] and the appearance of zona pellucida between the primary oocyte and GCs [6]. After puberty, most follicles gradually die via apoptosis and atresia [7]. In contrast, primary follicles escape death, transforming into secondary follicles (also known as dominant follicles), by the action of follicle-stimulating hormone (FSH) secreted by the hypothalamic–pituitary–ovarian axis. The dominant follicles begin to acquire their vascular system where monolayer GCs of these follicles proliferate into multiple layers. The secondary follicle transforms into the antral follicle as small, fluid-filled cavities gradually form within the follicle and eventually fuse into a larger single cavity [8]. In the antral follicle, the diameter of the primary oocytes is proportional to the diameter of the follicle [9], and the GCs differentiate into two subsets: cumulus cells (CCs) located around the oocyte and mural granulosa cells (MGCs) located outside the sinus [10] (Figure 1A).

#### 1.1.2. Oogenesis

The primary oocytes of most female mammals, such as humans, pigs and cattle, are chronically arrested in the diplotene stage of prophase of the first meiotic, regulated by a high concentration of cyclic adenosine monophosphate (cAMP), awaiting a signal to re-enter meiosis. The oocytes with a developed nucleus and highly loosened chromatin are called the germinal vesicle (GV) stage. During puberty, luteinizing hormone (LH) and FSH levels are physiologically increased, and oocytes resume meiosis by activating the maturation-promoting factor (MPF) through cAMP reduction and GV breakdown (GVBD). After the first division, the oocyte excludes the first polar body (PB1). It rapidly arrests in the metaphase II (MII) stage [11], which is released from the follicle by periodic hormonal regulation and awaits fertilization in the fallopian tube to complete meiosis [12]. Residual GCs are transformed into large luteal cells which produce progesterone to attract sperm efficiently, and residual follicular membrane cells are transformed into small luteal cells which secrete estrogens and androgens to regulate hormone balance [13]. When the sperm penetrates the oocyte, the oocyte extrudes the second polar body (PB2) [14] (Figure 1B).

#### 1.1.3. Development of the Zygote

After fertilization, the nuclei of the sperm and oocyte, called the paternal and maternal pronuclei (PN), are reset to a basal state of epigenetic modifications to support embryonic development into a new individual [15]. After several cell divisions, maternally derived mRNAs and proteins are removed and zygotic gene activation (ZGA) or what is more commonly known as embryonic genome activation (EGA) occurs, a process that has been represented as the maternal-to-zygotic transition (MZT), in which control of the developmental process is transferred from the maternally derived material to the newly synthesized products of the zygotic genome.

**Figure 2:**
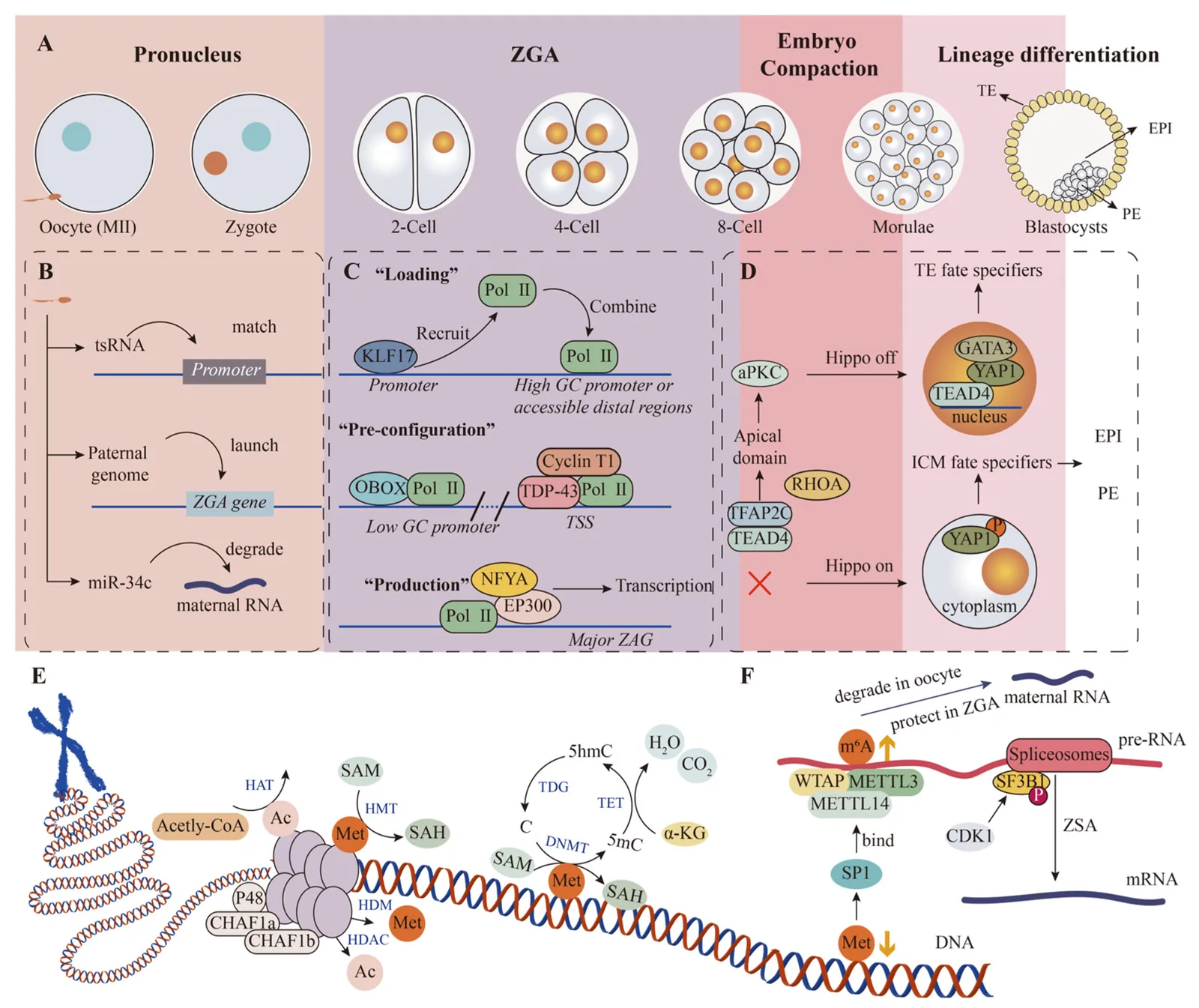
The process of zygote development in mammals. (**A**) Stages of zygote development. ZGA: Zygotic gene activation; TE: trophectoderm; PE: primitive endoderm; EPI: epiblast. (**B**) Effects of paternal genes. tsRNA: Small RNAs derived from sperm tRNAs. (**C**) Regulation of ZGA. KLF17: Krueppel-like transcription factor 17; Poll II: polymerase II; OBOX: PRD-like homeobox domain transcription factor; TDP-43: tar DNA-binding protein; TSS: transcription start site; EP300: E1A binding protein p300; NFYA: nuclear transcription factor Y subunit alpha. (**D**) Regulation of the first cell determination. TFAP2C: Transcription factor AP-2 gamma; TEAD4: TEA-domain family member 4; RHOA: ras homolog family member A; aPKC: atypical protein kinase C; GATA3: GATA binding protein 3; YAP1: yes-associated protein 1. (**E**) Chromatin accessibility regulates epigenetic modifications. CHAF: Chromatin assembly factor-1 subunit; H: histones; AC: acetylation; HAT: histone acetyltransferase; HDAC: histone deacetylases; Met: methylation; HMT: histone methyltransferase; HDM: histone demethylase; SAM: S-adenosylmethionine; SAH: S-adenosylhomocysteine; DNMT: DNA methyltransferases; TET: ten eleven translocation DNA; α-KG: alpha-ketoglutarate; TDG: thymine-DNA glycosylase DNA; C: unmodified cytosine; 5mC: 5-position of cytosine; 5hmC: 5-hydroxymethylcytosine. (**F**) RNA modulation. m6A: RNA N6-methyladenosine; WTAP: WT1-associated protein; METTL: N6-adenosine-methyltransferase; SP1: Sp1 transcription factor; SF3B1: splicing factor 3b subunit 1; CDK1: cyclin-dependent kinase 1; ZSA: zygotic splicing activation.

At the morula stage, the cells in the outer layer with apical polarity initiate trophectoderm (TE) differentiation. The embryo gradually forms a hollow sphere as the cells in the embryo undergo compaction. When the first cell fate decision occurs at the blastocyst stage, the outer layer cells exit from pluripotency and become TE cells [16]. The cells in the center of the blastocyst, called inner cell mass (ICM) cells, undergo the second cell fate determination and segregate into primitive endoderm (PE; a predecessor of the yolk sac) and epiblast (EPI; the progenitor for the fetus) with strong cell pluripotency [17]. Subsequent embryonic diapause (ED) at the blastocyst stage awaits the signal to implant in the uterus (Figure 2A).

Various metabolites and metabolism-related enzymes undergo intricately programmed changes during embryonic development, playing critical roles in numerous cellular events. Metabolism is a housekeeping function for cells and an active participant in regulating cell fate during development. However, it is crucial to recognize that varying physiological conditions dynamically regulate metabolic processes.

### 1.2. Cell Metabolism

#### 1.2.1. Carbohydrate Metabolism

Glycolysis, known as the Embden–Meyerhof–Parnas (EMP) pathway, produces pyruvate, lactate, and small amounts of ATP from carbohydrates under anaerobic conditions. Glycolysis under aerobic conditions is called aerobic glycolysis, also known as the ‘Warburg effect’, and is associated with high cell proliferation levels. Glycolysis is inefficient at producing ATP compared to mitochondria respiration. Still, the overall kinetics are orders of magnitude faster than the tricarboxylic acid cycle (TCA), making aerobic glycolysis the primary pathway for ATP synthesis.

The pentose phosphate pathway (PPP) has two main products, NADPH, which reduces oxidized glutathione (GSSG) to maintain reduced glutathione (GSH) levels, and ribulose 5-phosphate (R5P), which forms ribulose phosphate pyrophosphate (PRPP), an intermediate for de novo nucleotide and salvage synthesis of nucleic acids.

The hexosamine biosynthesis pathway (HBP) end product is UDP-N-acetylglucosamine (UDP-GlcNAc), which involves post-translational glycosylation modifications of specific proteins and is an essential substrate for the Leloir pathway for the synthesis of hyaluronic acid (HA) in vivo.

#### 1.2.2. Lipid Metabolism

Fatty acid (FA) metabolism is highly efficient and produces 3.5 times more ATP than glucose. FA beta-oxidation (FAO) is an essential catabolic pathway of FAs, which CPT regulates. Stearoyl CoA desaturase (SCD) governs the conversion of saturated and unsaturated FAs.

Triglycerides (TAGs), the primary lipid class in cells, are stored as lipid droplets (LDs) in cytoplasm. Phospholipids (PLs) are the most abundant lipids in eukaryotic cell membranes, and phosphatidylcholines (PCs), phosphatidylethanolamines (PEs), and sphingomyelins (SMs) are the structural units of functional membranes and determine the physicochemical properties of the majority of cell membranes. Cholesterol contributes to the synthesis of steroid hormones in the organism.

#### 1.2.3. Amino Acid Metabolism

Amino acid metabolism involves the interconversion of multiple amino acids and the production of specific metabolites. The serine–glycine–one-carbon (SGOC) pathway is a metabolic network comprising two core interconnected cycles: the folate and the methionine cycle. The trans-sulfuration pathway (TSP) entails the transfer of the sulfur atom of methionine to serine to produce cysteine, which could be utilized for GSH synthesis to protect against oxidative stress. The TSP also involves the γ-glutamine cycle and taurine metabolism. GSH produced by the γ-glutamine cycle provides antioxidant capacity. Numerous studies have shown that increasing antioxidant capacity contributes to oocyte and embryo development.

#### 1.2.4. Energetic Metabolism

The standard intermediate product of organismal metabolism, acetyl coenzyme A (CoA), enters the mitochondria to release H^+^ through TCA, which passes through oxidative phosphorylation (OXPHOS) in the electron transport chain (ETC) to generate ATP, which is used in biological activities of the organism.

In cytosol and mitochondria, isoenzymes NAD^+^ kinase1/2 (NADK1/2) catalyze the conversion of NADP(H) to NAD(H). Cytosolic NADPH is an essential substrate for the synthesis of FAs and GSH, while mitochondria NADPH is involved in many critical biosynthetic metabolisms.

In addition, specific metabolic processes involve altering the cellular redox state, such as the interconversion of pyruvate and lactate, α-keto acid as a substrate of lactate dehydrogenase (LDH) producing NAD^+^ and α-hydroxybutyrate, and the Aspartate (Asp)-malic acid (Mal) shuttle.

### 1.3. Purpose

The clarification of early embryonic development factors contributes to assisted reproductive technology (ART) to improve female fertility. It promotes in vitro production (IVP) in the agriculture of meat consumption and economic demand for livestock animals. However, the application of ART and IVP is greatly limited. Recently, methodological advances in bioinformatics and single-cell multi-omics technologies have provided approaches to get a glimpse of metabolic changes in early embryos [18], which on the one hand expands the scope of metabolic assays and on the other hand offers a new basis and perspective for the study of early embryos.

Although previous reviews have substantially advanced our understanding of energy metabolism during oocyte maturation and preimplantation development, most have focused on individual metabolic pathways, specific developmental stages, or the role of mitochondria in ATP production. The main focus of this review is therefore to provide an integrated view of how metabolic pathways and metabolites participate in key events of early mammalian embryonic development, including oocyte maturation, ZGA, compaction, and the first two lineage decisions. In particular, this review differs from previous reviews in three respects. First, it organizes metabolic changes around critical developmental transitions rather than around isolated pathways. Second, it integrates carbohydrates, lipids, amino acids, and energetic/redox metabolism and highlights metabolites not only as energy substrates but also as signaling molecules and substrates or cofactors for epigenetic modifications. Third, it distinguishes conserved mechanisms from species-specific observations in mice, humans, cattle, and pigs and discusses the implications for ART, embryo quality assessment, and IVP. By linking metabolism to developmental programs, this review aims to provide a new conceptual framework and research basis for understanding and optimizing early mammalian embryonic development. We also highlight future directions, including the integration of metabolomics, single-cell multi-omics, and functional validation.

## 2. Regulatory Factors of Embryonic Development

### 2.1. Maternal Environment

The oviduct is the site of the release of matured mammalian oocytes. It is also the physiological site of sperm capacitation, gamete fertilization and the early development of the embryo before entering the uterus. Therefore, physiological or pathological changes in the maternal environment may affect the oocyte maturation or fertilization process, thus incalculably impacting embryo development.

#### 2.1.1. Follicular Microenvironment

Single-cell transcriptome analysis of the human preovulatory follicles revealed the presence of six different cell types in the follicular fluid, including GCs, epithelial cells and immune cells such as macrophages, dendritic cells, T cells, and neutrophils, and GCs with high expression of adhesive-related molecules caused macrophage recruitment and residence, leading to heterogeneity of the immune cell proportion in different preovulatory follicles, which interacted with each other and were involved in the regulation of the inflammatory response and extracellular matrix remodeling to promote the resumption of oocyte meiosis [19]. Combined ultrafiltration and LC-MS/MS studies of human and mouse follicular fluid showed the presence of the highly conserved C3a peptide, a cleavage product of complement C3a, in the follicular fluid of mature oocytes, which promotes F-actin aggregation and spindle migration and has an essential impact on improving oocyte quality [20] (Figure 1C,D).

#### 2.1.2. Granulosa Cells (GCs)

The ability of oocytes to metabolize glucose is impaired by the deficiency of high-affinity insulin-regulated glucose transporter SLC2A4 (or GLUT4) and glycolysis rate-limiting enzyme phosphofructokinase (PFK) [21], making oocytes dependent on the uptake of other metabolic substrates. The glucose and lipid metabolism of GCs produces many energy substrates and signaling molecules, which are efficiently transported through various transport pathways established between the oocyte and GCs. The communication between oocytes and GCs is mainly achieved through the gap junction, the paracrine pathway, and the adhesion junctions and extracellular vesicle (EV)-mediated transport [22] (Figure 1E).

The CCs interact with the oocyte mainly through the non-contacting gap junction and adhesion junction of transzonal projections (TZPs) to form a structure called the cumulus–oocyte complex (COCs). TZPs are a type of filamentous pseudopodium formed by the CCs that effectively allow the physical transfer of metabolites (e.g., lactate and pyruvate), signaling regulatory factors (e.g., cAMP and cGMP), and oocyte maturation-associated RNAs between COCs to provide the energy and substances for oocyte development in the follicle or in vitro [23]. Loss of the connexin 43 (CX43) protein disrupts gap junctions between the oocyte and CCs, impairing the meiotic capacity of the oocyte, which causes impaired meiotic capacity of oocyte, suggesting that junction communication is essential for oocyte growth and meiosis [24].

The oocyte can regulate the metabolic pathways of CCs through the secretion of soluble paracrine factors such as bone morphogenetic protein 15 (BMP-15) and fibroblast growth factor 8 (FGF-8) [25]. Without an oocyte, CCs are not metabolically competent and metabolically driven processes. However, in response to oocyte demand, CCs exhibit elevated glycolytic activity and mRNA expression of the PFK and LDH [26].

A reduction in CC glucose uptake may lead to a decrease in oocyte ATP, which ultimately results in an impaired ability of oocyte meiotic maturation to the MII stage [27]. Wen’s study showed that pyruvate and lactate, glycolytic products of CCs, act as metabolic substrates that enter the oocyte via a monocarboxylate transporter (MCT) to promote oocyte maturation [28].

During the preovulatory period, HA synthesized by MGCs and CCs builds an extracellular matrix, facilitating CC expansion and ovulation and mediating sperm penetration into the oocyte during fertilization. MGCs secrete progesterone and estrogen to supply the hormones required for oocyte maturation.

Therefore, GCs are essential in maintaining oocyte meiotic arrest and regulating oocyte growth and development. Oocyte maturation requires metabolic synergy between the oocyte and GCs.

### 2.2. Embryonic

The quality of the embryo influences its developmental potential. There are many factors other than the maternal environment that contribute to poor embryo quality, such as the quality of the oocyte, which involves synchronized nuclear and cytoplasmic maturation of the oocyte, and differences in energy metabolism and oxidative stress due to the function of the mitochondria, the influence of paternal genes, and the influence of epigenetic changes in the embryo (Table 1).

#### 2.2.1. Maturation of the Oocyte

Oocyte quality reflects the intrinsic developmental potential of an oocyte. The synchronized maturation of the nucleoplasm of the oocyte has an essential impact on oocyte quality and fertilization. Nuclear maturation of the oocyte indicates the completion of the first meiotic division, while cytoplasmic maturation is required to accumulate mRNAs, proteins and substrates for subsequent fertilization and development. During in vitro maturation (IVM) of oocytes, cAMP concentration usually decreases after separation from the maternal environment, leading to premature maturation of the oocyte nucleus, which reduces the developmental competence of the oocyte [31].

In addition, the oocyte chromatin structure has been classified into two configurations according to the density of the chromatin and whether the nucleolus is surrounded by chromatin: the non-surrounded nucleolus (NSN), in which chromatin is scattered throughout the nucleolus region, and the surrounded nucleolus (SN), in which chromatin is condensed around the nucleolus [54]. The GV oocyte with SN-type chromatin configuration has lower transcription levels, facilitating subsequent maturation in multiple species [29,30], suggesting that the chromatin configuration at the GV stage is related to developmental competence. Inhibition of transcriptional activity in the GV stage accumulates the molecules and metabolic requirements necessary for the subsequent growth and development of oocytes to enhance oocyte developmental potential.

#### 2.2.2. Mitochondria

The primary function of mitochondria is OXPHOS, which produces ATP by transferring H^+^ of NADH and FADH2 through the inner mitochondria membrane. NADH and FADH2 are made by the products of glycolysis and FAO through the TCA.

Unlike somatic mitochondria, the mitochondria of immature oocytes have a rounded, hooded morphology, with few cristae and a less efficient capacity until the morula stage [55]. The number of mitochondria in the antral follicle oocyte is greater than in somatic cells and positively correlates with more significant embryo developmental competence [33].

Mitochondria distribution reflects the developmental potential of the embryo. During oocyte meiotic maturation, mitochondria accumulate around the spindle in response to the energy demands of mitosis, preventing spindle damage and chromosome misalignments [32,55]. In the cytoplasm of the mature oocyte, mitochondria are evenly distributed to promote uniform distribution of mitochondria in the blastocyst during early embryonic division [34].

The mitochondrial genome (mtDNA) undergoes degradation, rearrangement, mutations and a reduction in copy number occurring with the age of the individual [56]. In mouse oocytes, mitochondria damage has been associated with decreased ATP and increases reactive oxygen species (ROS), leading to impaired spindle formation and chromosomal misalignment, ultimately perturbing MII and subsequent embryo development. Further studies transferring the nucleus to an ooplasm containing healthy mitochondria restored MII and embryonic development [57], suggesting an effect of mitochondria on the nuclear genome.

#### 2.2.3. Influence of the Paternal Genes

Sperm tsRNAs, small RNAs derived from sperm tRNAs that preferentially match promoter regions of embryonic genes to regulate gene expression in the offspring, represent a paternal epigenetic factor that mediates the intergenerational inheritance of diet-induced metabolic disorders [58], and sperm-transmitted miR-34c may regulate preimplantation embryonic development by affecting maternal mRNA degradation [59].

Single-cell transcriptional analysis of androgenotes (AGs) in uniparental models showed a shorter cell division cycle in AG compared to parthenogenotes (PGs) or biparental human embryos. The development of the androgen gene was accompanied by the emergence of expression pathways related to cell differentiation, DNA-binding transcription, RNA biosynthesis and the RNA polymerase II (Pol II) transcriptional regulatory complex, and cell death. The significant overexpression of ZGA markers in the fourth cell cycle in AG compared with biparental embryos demonstrates the role of paternal genes in genome activation in zygotes and the involvement of the human paternal haploid genome in the early stages of embryonic development and its role in the transcriptome [60]. Recent studies have shown that human ZGA is initiated from the paternal genome launched at the eight-cell stage in biparental embryos [61]. This clarifies a critical time when the paternal genome influences the early embryo (Figure 2B).

### 2.3. Epigenetics

Many heritable changes in gene expression that are not DNA sequence changes occur in the developing oocyte or embryo, including epigenetic modification of the genome and histone, such as methylation, acetylation and alterations in chromatin accessibility, which mediate physiological processes such as oocyte maturation, ZGA and cell fate decision by regulating the spatiotemporal expression of target genes for reprogramming of the embryo. Even many of the previously mentioned modifiers are essentially epigenetic alterations.

With the development of technology, DNase-seq, ATAC-seq, NOMe-seq, ChIP-seq, CUT&RUN and CUT&Tag can be utilized to obtain high-resolution maps for chromatin accessibility and epigenetic modifications.

#### 2.3.1. Transcriptional Activity and Chromatin Accessibility

Chromatin accessibility refers to the extent to which DNA binds to factors, including transcription factors and epigenetic modifiers, representing a region’s potential to regulate gene expression. Overall, the number of accessible regions identified in the genome gradually increases in embryonic development and is enriched for promoters, CpG islands and distal regulatory elements such as enhancers.

##### Transcription Factors (TFs)

A study that developed small-scale Tn5-assisted chromatin cleavage with sequencing (Stacc-seq) showed that during the transition from the minor to the major ZGA, RNA Pol II in mouse embryos undergoes ‘loading’, meaning that Pol II is preferentially loaded into CG-rich promoters or accessible distal regions, ‘pre-configuration’ leading Pol II to relocate in future gene targets before genome activation, and ‘production’ result in Pol II undergoing complete transcription in the major ZGA. In this process, inhibition of the minor ZGA impairs Pol II pre-configuration and embryonic development [62].

In two-cell embryos, maternal KLF17 binds to the promoter and recruits Pol II [63], OBOX regulates the ‘pre-configuration’ of Pol II to binding in low-GC promotion [64], and TDP-43 interacts with Cyclin T1 co-regulating Pol II to enrich around the TSS of major ZGA genes, together regulating genome activation [65]. Ep400 is a chaperone of H3.3, which forms protein complexes with NFYA, regulates H3.3 distribution between euchromatin and heterochromatin, and promotes transcriptional elongation of ZGA genes that regulate Mitochondria functions and rate-limiting enzymes of the TCA [66]. Recent studies have shown that a deficiency in the regulatory factors TCF3 and TCF12 impair the activation of key oocyte genes and folliculogenesis [67] (Figure 2C).

In human, cow and mouse morula embryos, there is a conserved TE placental progenitor program whereby Hippo signaling elements initiate the nuclear expression of the TE-associated factor GATA3 [68]. Atypical protein kinase C (aPKC) is activated when outer cells acquire an apicobasal cell polarity, which affects the nuclear expression of yes-associated protein 1 (YAP1), a cofactor of the Hippo signaling transcription factor TEA-domain family member 4 (TEAD4), to ensure nuclear co-localization of GATA3 and YAP1 in outer cells, thereby initiating TE segregation in the morula [68]. It was shown that TFAP2C-TEAD4-RHOA formation in 2–8 cells establishes a bistable switch in the Hippo pathway that regulates the first fate decision of the cell, based on the presence or absence of the apical structural domain; when the cell possesses the apical structural domain, the Hippo pathway is inhibited and the cell differentiates towards TE, while the absence of the apical structural domain activates the Hippo pathway and the cell moves towards ICM differentiation [69] (Figure 2D).

##### Chromatin Assembly

The chromatin-assembly factor-1 (CAF-1) complex is a trimeric histone chaperone composed of three subunits, CHAF1a, CHAF1b, and p48, which deposits histones H3 and H4 onto replicated DNA during chromatin assembly. The chromatin assembly factor-1 subunit b (CHAF1b) mediates the replacement of H3.3 with H3.1/3.2, leading to a decrease in inhibitory histone labeling (H3K9me2/3 and H3K27me2/3) and an increase in active histone labeling (H3K4me2/3) (Figure 2E). It has been shown that CHAF1b depletion alters chromatin accessibility of lineage-specific genes and epigenetic modifications genes (e.g., histone acetyltransferase Ep300 and Kat6a), leading to cell apoptosis and down-regulation of the expression of key regulators of cell fate specification (including Oct4, Cdx2, Sox2, and Nanog) [70].

#### 2.3.2. DNA Methylation

DNA methylation reduces genome transcription, and extensive demethylation of the biparental genome occurs before the first cleavage, except in imprinting control regions (ICRs) and some retrotransposons [71]. Demethylation of the paternal genome is more rapid and active by the substituting histones with protamines [72]. The maternal genome resists the initial wave of demethylation instead of passive demethylation dependent on DNA replication in subsequent cleavages, resulting in epigenetic asymmetry in the early embryo [36].

DNA methyltransferases (DNMTs) and ten-eleven translocation (TET) regulate DNA methylation levels [73]. Incomplete DNA demethylation during early embryonic development reduces ZGA, leading to delayed embryonic development [37]. Recent studies have shown that maternal Pramel15 targets DNMT1 for degradation via Cullin-RING E3 ligases, thereby increasing zygote DNA demethylation [38].

At the morula and blastocyst stages of embryonic development, DNMTs remodel de novo DNA methylation, which enhances embryonic stem cell (ESC) transition from naive to primed pluripotency [39]. Until the blastocyst stage, when the first cell fate decision occurs, DNA methylation is re-established in the outer layer cells [40], which exit from pluripotency and become TE cells. High levels of DNA methylation at this time repress the transcription of multiple retrotransposons and autosomal imprinting [41,42], and regulate placental development and function [43], which is critical for embryo survival (Table 1).

#### 2.3.3. Histone Modification

Histone modifications are key regulatory events that affect the interactions of transcriptional regulators with chromatin throughout preimplantation embryogenesis. Histone methylation is regulated by histone methyltransferase (HMT) and histone demethylase (HDM). The level of histone acetylation is regulated by histone acetyltransferase (HAT) in conjunction with histone deacetylase (HDAC). These enzymes are closely related to transcriptional status dynamically during specific periods of embryonic development [74,75] and late advances as follows (Table 1).

##### Histone Methylation

H3K4me3 is a marker for active promoters, and a non-canonical form of H3K4me3 (ncH3K4me3) is present in CpG regions of mature mouse oocytes, which overlaps almost exclusively with partially methylated DNA domains [45] and is replaced by H3K4me3 until the major ZGA [46]. Unlike for mice, H3K4me3 is present in human GV and MI oocyte promoters [44]. After fertilization, H3K4me3 is rapidly depleted in the paternal genome and re-established during the major ZGA [76]. These results of the change in dynamic H3K4me3 correspond to changes in DNA methylation levels during embryonic development.

There is a negative correlation between H3K27me3 with DNA methylation and H3K4me3 [76] in mouse MII oocytes [77] and preimplantation embryos [47]. H3K27me3 is an imprinted marker of the maternal Xist gene, which regulates paternal allele-specific imprinted X chromosome inactivation (XCI) in mouse oocytes from growth to preimplantation embryo [78]. Removing this imprint results in maternal Xist expression and maternal abnormality of X chromosome inactivation in preimplantation embryos [79]. H3K27me3 in normally fertilized two-cell mouse embryos exhibits asymmetric markers globally erased in the paternal genome and selectively depleted in the maternal genome [48,80]. In contrast, at the human ZGA stage, it is comprehensively lost in the biparental genome [44]. At the mouse blastocyst stage, the H3K27me3 imprinting is diluted in ICM and is lost in the EPI at E6.5 embryo [45]. When H3K27me3 is missing from blastocysts in mice, although it does not affect the continued development of the blastocysts, it causes post-implantation embryonic arrest. Studies have shown this may be involved in the development of the placenta [81].

H3K9me3 regulates heterochromatin formation and function to maintain genomic stability [82]. Commonly, asymmetric demethylation of H3K9me3 occurs in the parental genome of fertilized embryos [49], which would otherwise result in ZGA failure [50] by inhibiting long terminal repeat (LTR) retrotransposon transcriptional activity [83]. In mouse post-implantation embryos, H3K9me3 is reconstituted to repress the expression of repeat elements and lineage-specific protein genes [49].

##### Histone Acetylation

H3K27ac is established at the pronucleus stage and enriched in promoters, introns, and intergenic regions. H3K27ac is enriched in the secondary ZGA promoter at the two-cell stage, with a rapid loss at the mid- to late two-cell stage and a gradual increase after the four-cell stage [51]. CBP/p300 acetyltransferase deposits H3K27ac onto open condensed chromatin and ensures ZGA. In contrast, HDACs deacetylate the H3K27ac domains to prevent premature expression of ZGA. Coordinated activities of CBP/p300 and HDACs maintain the transition of H3K27ac from the oocyte stage to the preimplantation stage [84]. Dynamic studies of histone acetylation during embryonic development show that humans and mice have a broad H3K27ac structural domain that overlaps with a broad H3K4me3 structural domain before ZGA and a typical H3K27ac peak at ZGA, the amount of which is dynamically regulated by p300 [85].

##### Non-Classical Histone Modifications

Histone crotonylation is a widespread non-acetyl histone lysine modification, but its physiological functions remain almost unknown. It has been shown that during the differentiation of human and mouse ESCs, endodermal cells induce the crotonyl-CoA-producing enzymes, which increases histone crotonylation and the expression of endodermal genes [52].

**Figure 3:**
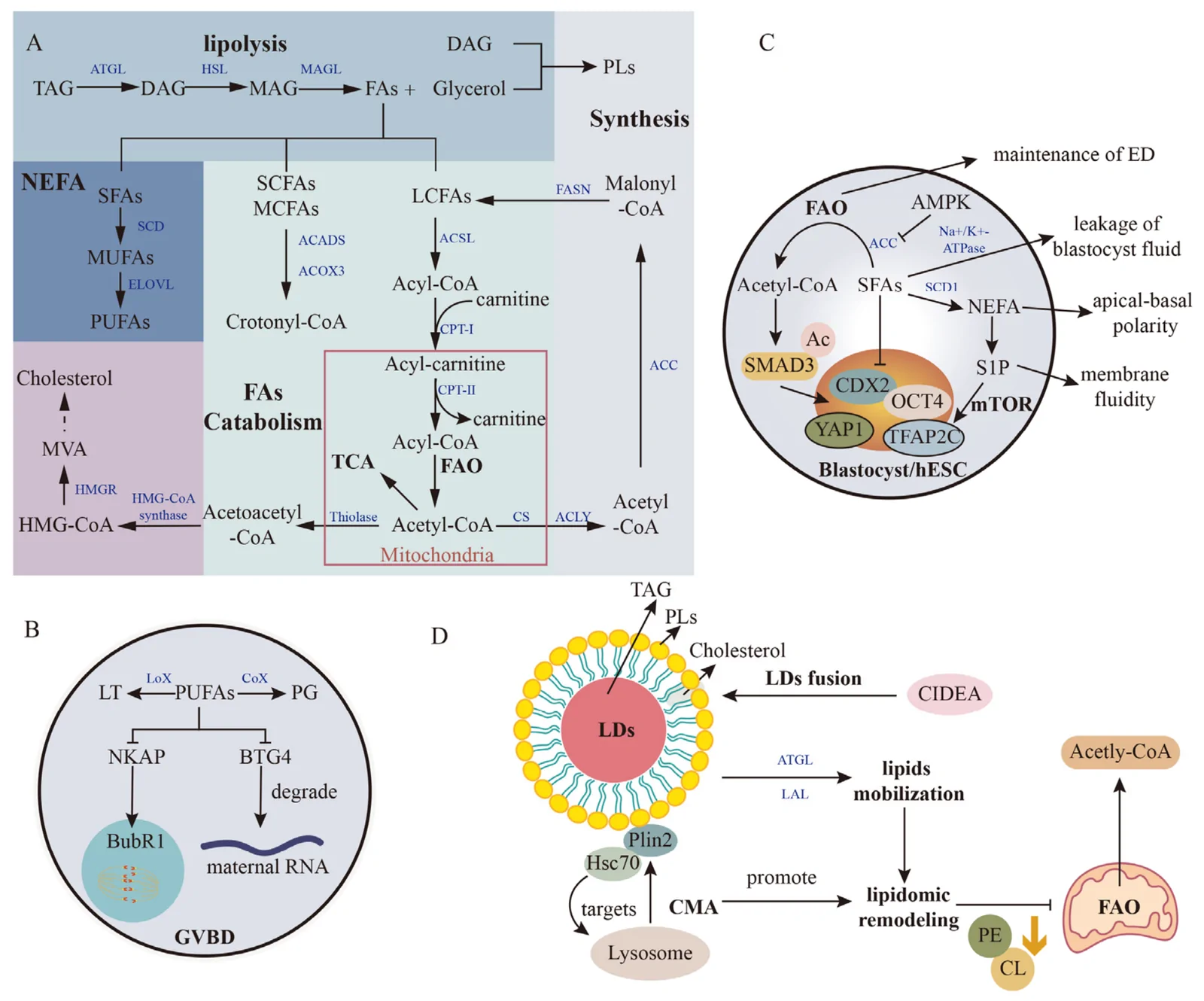
Important lipid metabolism pathways during early embryonic development. (**A**) Lipid metabolism. TAG: Triacylglycerol; ATGL: adipose triglyceride lipase; DAG: diacylglycerol; HSL: hormone-sensitive lipase; MAG: monoacylglycerol; MAGL: monoacylglycerol lipase; FAs: fatty acids; PLs: phospholipids; SFAs: saturated fatty acids; SCD: stearoyl CoA desaturase; MUFAs: monounsaturated fatty acids; ELOVL: elongases of very long-chain fatty acids; PUFAs: polyunsaturated fatty acids; NEFA: unsaturated fatty acid; LCFAs: long-chain fatty acids; ACSL: long-chain acyl-CoA synthetase; CPT: carnitine palmitoyl-transferase; CS: citrate synthase; ACLY: ATP citrate lyase; ACC: acetyl-CoA carboxylase; FASN: fatty acid synthase; SCFAs: short-chain fatty acids; MCFAs: medium-chain fatty acids; ACADS: mitochondria short-chain acyl CoA dehydrogenase; ACOX3: peroxisomal acyl-CoA oxidase; HMG-CoA: 3-hydroxy-3-methylglutaryl CoA; HMGR: HMG-CoA reductase; MVA: mevalonic acid. (**B**) Role of PUFAs in GVBD oocytes. LT: leukotrienes; LoX: lipoxygenase; PG: prostaglandins; CoX: cyclooxygenase; NKAP: NFKB-activating protein; BTG4: B-cell translocation gene 4; BubR1: Bub1-related kinase. (**C**) Role of lipid metabolism in blastocyst/hESCs. ED: Embryonic diapause; SMAD: Sma and mad-related protein; Ac: acetylation; OCT4: octamer binding transcription factor; S1P: sphingosine-1-phosphate. (**D**) The metabolism and regulation of LDs. LDs: Lipid droplets; CIDEA: cell death-inducing DFFA-like effector A; LAL: lysosomal acid lipase; Plin2: perilipin 2; Hsc70: heat shock cognate protein 70; CMA: chaperone-mediated autophagy; PE: phosphatidylethanolamines; CL: cardiolipins.

Crotonate (NaCr) is a short-chain fatty acid (SCFA) precursor of crotonyl-CoA. ACADS and ACOX3 catalyze the oxidation of short-chain fatty acid acyl-CoA and branched-chain fatty acids (Figure 3A), and knockdown of these enzymes reduces explicitly intracellular crotonyl CoA levels. This result directly links short-chain fatty acid oxidation to histone crotonylation during endodermal differentiation, implicating histone crotonylation as a metabolically sensitive epigenetic modification, further explaining the role of the metabolic switch from glycolysis to oxidative phosphorylation in promoting endodermal gene expression during the period after lineage differentiation [52].

Recent studies have shown that p300 also catalyzes site-specificity in the crotonylation of histones, including H3K18cr, H3K9cr, H3K23cr, H3K36cr, H3K27cr, and H3K14cr. They have also demonstrated that transcription and expression of the embryonic genome are positively regulated through H3K18cr, thus ensuring the successful progression of embryonic development to the blastocyst stage [53].

#### 2.3.4. RNA Modulation and Post-Transcriptional Regulation

N6-methyladenosine (RNA m6A) is one of the most abundant RNA modifications in eukaryotic mRNAs, regulated by the RNA m6A methyltransferase complex comprising METTL3, METTL14 and WTAP. Numerous studies have shown that RNA m6A modifications mainly reduce mRNA stability [86]. In mESCs, Mettl3 has been shown to induce stem cell pluripotency [87] and enhance transcriptional dormancy required for diapause [88] by promoting RNA m6A modifications.

Recently, single-cell m6A sequencing (scm6A-seq) revealed changes in m6A dynamics during development from the oocyte to the early embryo. The results confirmed that there are different mechanisms of m6A-regulated RNAs in the oocyte and early embryo, with m6A mediating RNA degradation in GV oocytes while protecting maternal RNAs during MII-to-zygote transition, which is likely because most of the RNAs involved in ZGA were protein-coding RNAs. In contrast, maternal RNAs contain a more significant proportion of non-coding RNA [89]. m6A modification may thus be involved in the mRNA regulation of transcription factors. A recent porcine somatic cell nuclear transplantation experiment confirmed that low DNA methylation improves the accessibility of the transcription factor SP1 to bind METTL14 promoter and promotes RNA m6A modification [90], which suggests crosstalk between RNA m6A and DNA methylation.

Alternative splicing is a prominent post-transcriptional RNA processing mechanism that forms different mRNA isoforms by regulating the combination of splice sites of the spliceosomes and precursor RNA (pre-RNA) through splicing factors. Recent studies have shown that SF3B1 is a crucial splicing factor, dependent on CDK1 phosphorylation activation, that regulates the zygotic splicing activation (ZSA) process in early embryonic development. This process coincides with the major ZGA and is vital for transitioning from totipotency to pluripotency in embryos [91] (Figure 2F).

## 3. Metabolic Homeostasis in Embryonic Development

Metabolic programs are regulated in time and space by regulators such as specific transcription factors or enzymes. Still, it is noteworthy that metabolic pathways also regulate specific biological processes such as oocyte maturation, gene expression, and epigenetic and post-translational modifications during embryonic development. Appropriate metabolic activity endows embryos with maximum developmental capacity by providing the energy required for embryonic development and supplying many substances that regulate embryo development. These atypical metabolic signaling functions establish unexpected and direct links between seemingly distant cellular processes. However, the metabolic alterations in embryonic development are dramatic and complex, and limited detection techniques in the past have led to a partial and fragmented understanding of metabolic processes during embryonic development. Furthermore, because metabolic phenotypes are strongly influenced by species, developmental stage, tissue context, culture conditions, and experimental models, metabolic changes observed in one system should not be generalized automatically to all mammals. Throughout this review, findings supported across multiple mammalian species are discussed as broadly conserved, whereas findings identified in a single species or in stem-cell models are explicitly presented as species- or model-specific observations.

Next, we will introduce common metabolic pathways, products and kinases (Table 2) and discuss how metabolism is linked to embryo developmental programs through its bioenergetic and metabolic signaling functions. This new insight helps to integrate metabolic patterns with upstream regulatory circuits, revealing the potential causal roles for metabolism in different periods of embryonic development.

### 3.1. Glucose Metabolism

**Figure 4:**
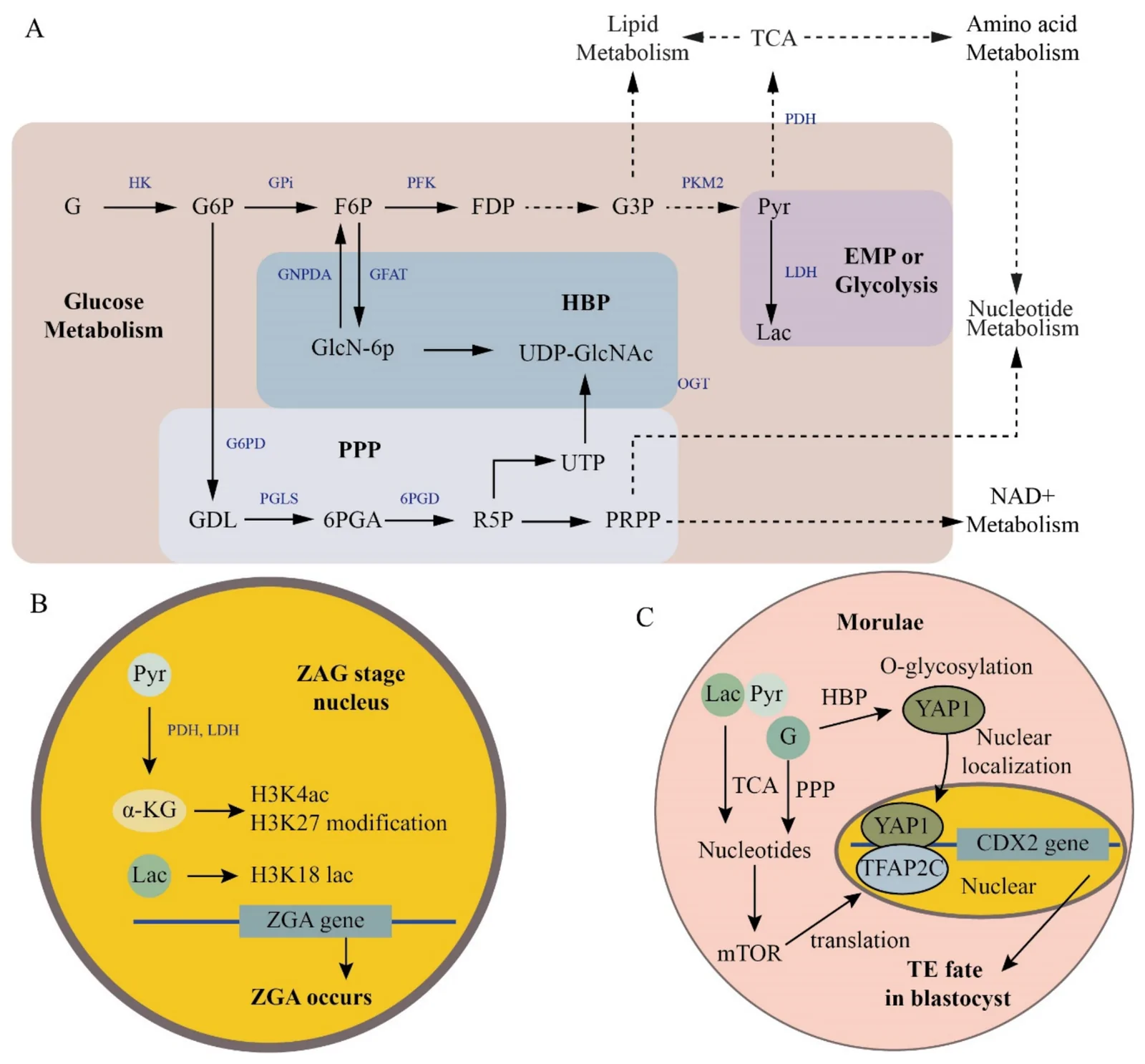
Important glucose metabolism pathways during early embryonic development. (**A**) Glucose metabolism. EMP: Embden–Meyerhof–Parna pathway or glycolysis; HBP: hexosamine biosynthesis pathway; PPP: pentose phosphate pathway; G: glucose; HK: hexokinase; G6P: glucose-6-phosphate; GPi: glucose-6-phosphate isomerase; F6P: fructose-6-phosphate; PFK: phosphofructokinase; FDP: fructose-1,6-diphosphate; G3P: 3-phosphoglycerate; PKM2: pyruvate kinase isozyme 2; Pyr: pyruvic acid; LDH: lactate dehydrogenase; Lac: lactic acid; GFAT: fructose-6-phosphate aminotransferase; GNPDA: glucosamine-6-phosphate deaminase; GlcN-6p: glucosamine-6-phosphate; UDP-GluNAc: uridine diphosphate N-acetylglucosamine; OGT: O-linked N-acetylglucosamine transferase; G6PD: glucose-6-phosphate dehydrogenase; GDL: gluconolactone; PGLS: 6-phosphogluconolactonase; 6PGA: 6-phosphogluconic acid; 6PGD: 6-phosphogluconate dehydrogenase; R5P: ribose 5-phosphate; PRPP: 5-phosphoribosyl-1-pyrophosphate; UTP: uridine triphosphate; TCA: tricarboxylic acid cycle. Black solid lines represent metabolic pathways, and black dashed lines represent partially omitted metabolic pathways. (**B**) Regulation of glucose metabolism during the ZGA stage. α-KG: alpha-ketoglutarate. (**C**) Regulation of glucose metabolism during the embryonic stage in morula. mTOR: mammalian target of rapamycin; YAP1: yes-associated protein 1; TFAP2C: transcription factor AP-2 gamma; CDX2: caudal type homeobox 2. In blastocysts, saturated fatty acids (SFAs) disrupt the localization of appropriate blastocyst-forming genes during preimplantation development in mice. In contrast, unsaturated fatty acids (NEFAs) are protective and promote the expression of blastocyst-specific proteins and cell fate [122]. In buffalo, it was also found that higher levels of FAs degradation and NEFAs contributed to blastocyst survival [123]. In hESCs, enhanced FAO contributes to acetyl-CoA accumulation, which enhances the acetylation and nuclear localization of SMAD3 to guide endoderm differentiation [124] (Figure 3C). Additional studies have illustrated that FOXO1 regulates the FAO metabolite levocarnitine, thereby promoting FAO, which contributes to the maintenance of the embryo in embryonic diapause before implantation [125]. These findings suggest that FAs catabolism may contribute to blastocyst survival and implantation-related processes.

Glucose is an important metabolite for ATP production by the COCs, and since glucose is difficult for oocytes to take up, nearly all the consumed glucose is metabolized in the CCs [100]. However, supplementation of pyruvate, a product of the glycolysis of glucose, failed to fully restore cumulus-denuded oocyte damage [27], suggesting that glucose plays a role in non-metabolic pathways in addition to ATP production. For example, a labeling experiment on glucose carbon illustrated the role of glucose in nucleotide production, where glucose at the two-cell stage is retained until the blastocyst stage for the synthesis of ribose for nucleotides, which is then used to synthesize the nucleobase portion of purine nuclei through the then-required endogenous Gly [101]. The major glucose metabolic pathways involved in these processes are summarized in Figure 4A.

#### 3.1.1. Glycolysis

Consistent with the oocyte, the activity of the rate-limiting glycolysis enzyme, PFK, is low in two-cell mouse embryos, thus limiting the glycolytic capacity of early embryos [102]. Although the above studies have shown that glycolysis remains in a low-activity state from the beginning of the oocyte to the two-cell embryo, it has long been demonstrated that pyruvate is essential for development beyond this stage. Its absence reduces O-glycosylation as well as the nuclear localization of pyruvate dehydrogenase (PDH), which diminishes H3K4 and H3K27 acetylation and H3K27 methylation in the ZGA, which leads to blocking the two-cell embryo [103] (Figure 4B). The authors further demonstrated that alpha-ketoglutarate (α-KG) mitigates the damage caused by pyruvate deficiency and allows embryos to develop to the blastocyst stage [103]. It is important to note that α-KG did not restore the changes in energy or redox balance caused by pyruvate deficiency, suggesting that pyruvate metabolism involves regulating redox capacity and energy metabolism.

Recent studies have found that lactate has an essential effect on ZGA activation. Using the recently redeveloped lactate sensor FiLa, Li et al. showed that lactate has been shown to promote lactylation of H3K18 in the two-cell mice embryos and human eight-cell embryos, which is the period of major ZGA activation, facilitating Pol II loading to encourage expressions of major ZGA genes [104]. Recent studies show that glycolysis-dependent lactylation affects even neural crest cell migration in post-implantation embryos [105].

Relative to primed hESCs, naive hESCs exhibit increased glycolytic flux and pluripotency, consistent with the ICM of human blastocysts, and the rate of glycolysis decreased after cell differentiation [106,107]. In vitro studies of PSC fate decisions demonstrate that metabolic switching is germ-layer-specific and controlled by the dynamics of specific transcription factors.

#### 3.1.2. Pentose Phosphate Pathway (PPP)

Past studies have shown that NADPH alleviates Xenopus oocyte apoptosis by resisting phosphorylation of caspase-2 by calcium/calmodulin-dependent protein kinase II (CaMKII) [108]. The PPP is an antioxidant production pathway that neutralizes ROS damage [109] and regulates intracellular redox status. However, only a tiny proportion of glucose is metabolized via the PPP pathway before oocyte maturation [21], suggesting that the oocyte is not entering a state of active energy metabolism. In addition, PRPP is also an intermediate in the de novo and rescue synthesis pathways of NAD^+^ [99]. Recent studies have shown that increasing NAD^+^ restores oocyte quality and fertility in aged mice by maintaining sirtuin activity [110].

Chi et al. reported that the PPP pathway has an important role in developing mouse eight-cell embryos into blastocysts. Firstly, the authors followed the flow of labeled carbon to other metabolites by U-13C labeling of glucose and pyruvate carbon atoms. The results determined that the substrate enters the TCA to provide energy from pyruvate, while glucose enters the PPP pathway to produce R5P. Secondly, it was found that R5P promotes the formation of uridine triphosphate (UTP) to produce UDP-GlcNAc, which forms a crosstalk with the HBP pathway. Finally, the study also confirmed that PPP may promote post-transcriptional modification of the TFAP2C protein by activating the mTOR signaling pathway, thereby increasing the transcription factor CDX2, a marker molecule for TE [111] (Figure 4C).

#### 3.1.3. Hexosamine Biosynthesis Pathway (HBP)

In COCs, the HBP pathway contributes to the expansion of cumulus cells through the synthesis of HA [112], whereas excessive HBP activity glycosylates heat shock protein 90 (HSP90) and reduces the developmental competence of mouse oocytes [113]. Recent studies have pointed out that excessive O-linked glycosylation in bovine COCs significantly reduces fertilization by affecting sperm penetration, sperm head decondensation, and pronuclear formation [93]. Thus, appropriate HBP activity facilitates oocyte extrusion at ovulation, transport to the site of fertilization, and sperm penetration.

In addition, O-linked glycosylation is also involved in the nuclear transport of certain cytosolic proteins [114], and it has been shown that reduced O-linked glycosylation inhibits the nuclear localization of PDH and epigenetic modification ability, leading to the blocking of ZGA in mice [103]. Secondly, UDP-GlcNAc enables O-linked glycosylation of YAP1 proteins at 1–4 specific loci and induces nuclear localization of YAP1 in pre-TE [111], which in turn expresses CDX2 and differentiates the cell to TE. Thus, the HBP pathways are also implicated in blastocyst formation by controlling the signaling and transcriptional events that underlie TE cell fate (Figure 4C).

Recent studies have shown that O-linked glycosylation increases glucose uptake of endometrial cells by glucose transporter 1 (GLUT1). These processes regulate the flow of glucose metabolism towards PPP and HBP in endometrial cells to influence embryo implantation [115]. The endometrial luminal epithelium is covered with highly glycosylated mucin that regulates blastocyst adhesion during implantation to modulate implantation, trophoblast functions and maternal–fetal immunotolerance [116].

### 3.2. Lipid Metabolism

Lipids are a significant form of energy storage and may act as signaling molecules that regulate a range of key biological processes. The importance of lipid metabolism on embryonic developmental competence is now beginning to be explored [117].

#### 3.2.1. Fatty Acids (FAs)

Several studies have shown that FAO is required to progress meiosis to the MII stage in mice [118,119] and bovine [120], which may lead to a decrease in FAs levels. A recent study by Li et al. confirmed that a decrease in polyunsaturated fatty acids (PUFAs) content during meiosis in mice helps to increase Nkap levels, prevents chromosomal misalignment, and contributes to BTG4-triggered oocyte mRNA decay [121] (Figure 3B). However, a recent study in pigs found that the content of FAs declined dramatically in porcine oocytes that began meiosis, while the level of FAO also decreased, suggesting that lipids in oocytes enter other lipid metabolic pathways [96].

#### 3.2.2. Lipid Droplets (LDs)

LD content increases at the morulae and decreases at the blastocyst stage in bovine embryos [95,126]. More detailed studies in mice have shown that LDs are synthesized in the endoplasmic reticulum of mouse oocytes as early as the follicular stage of development through the uptake of exogenous fatty acids [127]. Follow-up studies have confirmed that removing LDs does not affect fertilization or development of mouse MII oocytes to the blastocyst stage [128]. Because a large number of small and dispersed LDs are synthesized in the mouse morula embryo during development into a blastocyst, these small LDs tend to aggregate and fuse. It has been shown that the LD surface protein CIDEA transiently enlarges the LDs. As development proceeds, the LDs are lost in the epiblast and retained only in the TE. The enlarged LDs prevent LD degradation by cytosolic lipases during cell polarization, thus allowing LDs to mobilize into lysosomes at the onset of lumen formation [129].

The Hsc70-recognized LDs-associated protein Plin2 promotes the hydrolysis of LDs by ATGL through chaperone-mediated autophagy (CMA) degradation, which induces a decrease in cardiolipins (CLs) and PEs and an increase in PCs. Since CLs and PEs are significant components of mitochondria phospholipids essential for maintaining mitochondrial structure and function, CMA-induced lipid remodeling triggers the loss of mitochondrial cristae structure and impaired FAO, which reduces acetyl-CoA and acetylation levels of pluripotency genes, ultimately inducing mESCs to exit from pluripotency [130] (Figure 3D). LDs content is positively correlated with its species-specific embryonic diapause [131]. These studies demonstrate that TE cells of blastocysts are required to be enriched with sufficient LDs to provide energy for implantation and embryo survival. In contrast, LD degradation is responsible for influencing the exit of blastocysts from pluripotency.

#### 3.2.3. Cholesterol and Phospholipids

The elevated steroid hormone content of mature porcine oocytes, especially pregnenolone and progesterone, suggests that the dynamics of lipid metabolites may play a crucial role in nutrient regulation, hormone regulation and cellular homeostasis [96].

Recently, Zhang et al. investigated the characterization of lipids during embryonic development in mice and humans using an isopropanol-based extraction method, which recovers polar lipids such as phospholipids with good efficiency, and the Bligh & Dyer method, which recovers both polar phospholipids and neutral lipid, followed by combined high-performance liquid chromatography–mass spectrometry (HPLC-MS) analysis [94]. The results showed that PEs and phosphatidylinositols (PIs) were abundant in oocytes and decreased after fertilization; PCs and long-chain fatty acids (LCFAs) decreased after fertilization and increased in blastocysts; SMs are abundant in blastocysts; CLs are specifically increased only at the 2–4-cell stage. They showed that the dramatic lipid remodeling that occurs at the blastocyst stage involves an increase in the unsaturation of phospholipids, and SCD1 increased lipid unsaturation of phospholipids, which contributes to cytoplasmic membrane fluidity and apical–basal polarity, thereby promoting blastocyst development [94]. The sphingolipid pathway also regulates mTOR to control TFAP2C, thereby affecting the TE marker molecule, CDX2 expression and embryonic differentiation [111] (Figure 3C). The phospholipid composition and the extent of unsaturation substantially impact membrane fluidity [132,133]. Phospholipid-derived mediators such as, for example, endocannabinoids, lysophosphatidic acid and sphingosine-1-phosphate, are associated with endometrial receptivity, embryo spacing and decidualization based on evidence from both animal and human studies [134].

### 3.3. Amino Acid Metabolism

**Figure 5:**
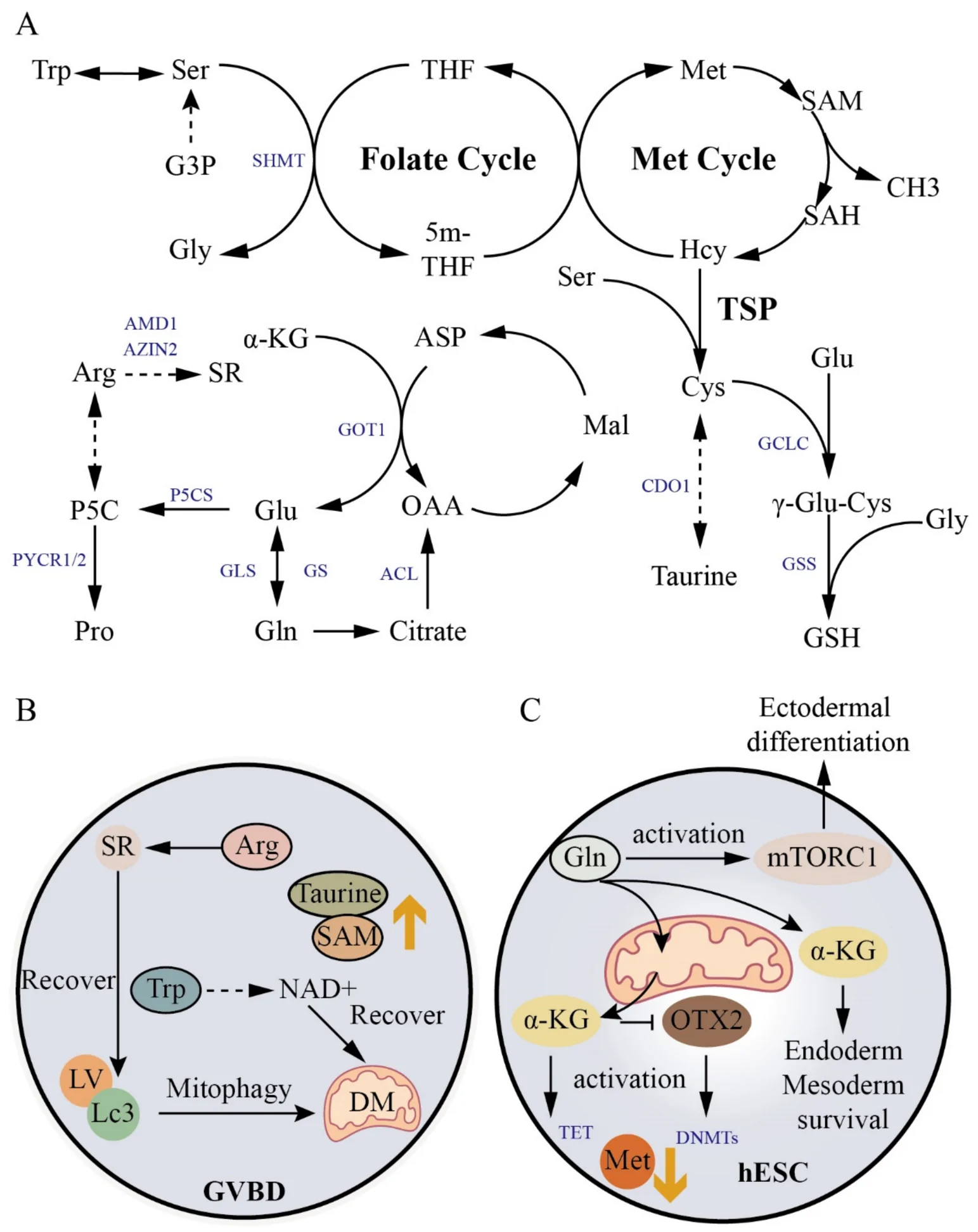
Important amino acid metabolism pathways during early embryonic development. (**A**) Amino acid metabolism. Trp: Tryptophan; Ser: serine; G3P: 3-phosphoglycerate; Gly: glycine; SHMT: serine hydroxy methyltransferase; THF: tetrahydrofolate; 5m-THF: 5-methy tetrahydrofolate; Met: methionine; SAM: S-adenosylmethionine; SAH: S-adenosylhomocysteine; CH3: methyl; Hcy: homocysteine; Cys: cysteine; Glu: glutamate; GCLC: glutamate–cysteine ligase catalytic; γ-Glu-Cys: γ-glutamyl–cysteine; GSS: glutathione synthetase; GSH: glutathione; Gln: glutamine; GS: glutamine synthetase; GLS: glutaminase; ACL: ATP-citrate lyase; OAA: oxaloacetate; Mal: malic acid; Asp: aspartate; α-KG: alpha-ketoglutarate; GOT1: glutamic-oxaloacetic transaminase 1; MDH1: malate dehydrogenase; P5CS: pyrroline-5-carboxylate synthase; P5C: 1-pyrroline-5-carboxylate; PYCR1/2: pyrroline-5-carboxylate reductase 1/2; Pro: proline; Arg: arginine; AZIN2: antizyme inhibitors 2; AMD1: adenosylmethionine decarboxylase 1; SR: spermidine. (**B**) Role of amino acid metabolism in GVBD oocytes. LV: lysosome vesicles; Lc3: microtubule-associated protein 1A or 1B light chain 3; DM: dysfunctional mitochondria; NAD^+^: nicotinamide adenine dinucleotides. (**C**) Role of amino acid metabolism in hESCs. mTORC1: mammalian target of rapamycin complex 1; OTX2: orthodenticle homeobox 2; TET: ten-eleven translocation; DNMTs: DNA methyltransferases.

Recent studies have shown that embryos have constitutive macropinocytotic protein uptake and digestion traits. These traits autonomously take up proteins deposited in oocytes or exogenous proteins released from dying cells within the blastocyst via lysosomes, demonstrating amino acid independence [135]. An overview of this amino acid metabolism is presented in Figure 5A.

#### 3.3.1. Catabolic Metabolism

Arginine (Arg) has several metabolic pathways in vivo [136] that produce nitric oxide (NO), urea, ornithine, citrulline, creatine, guanidine, glutamate, proline (Pro) and polyamines. In mature porcine oocytes, the levels of Arg and Pro are reduced, and their catabolism is enhanced [96]. Recent studies have shown that one of its products, spermidine supplementation, has been reported to improve oocyte quality, potentially through modulation of mitochondrial autophagy [137]. Spermidine supplementation enhances mitochondrial autophagy in oocytes from aged mice by restoring the co-localization pattern between the autophagosome marker microtubule-associated protein 1A or 1B light chain 3 (LC3) foci and lysosome vesicles (Figure 5B).

**Figure 6:**
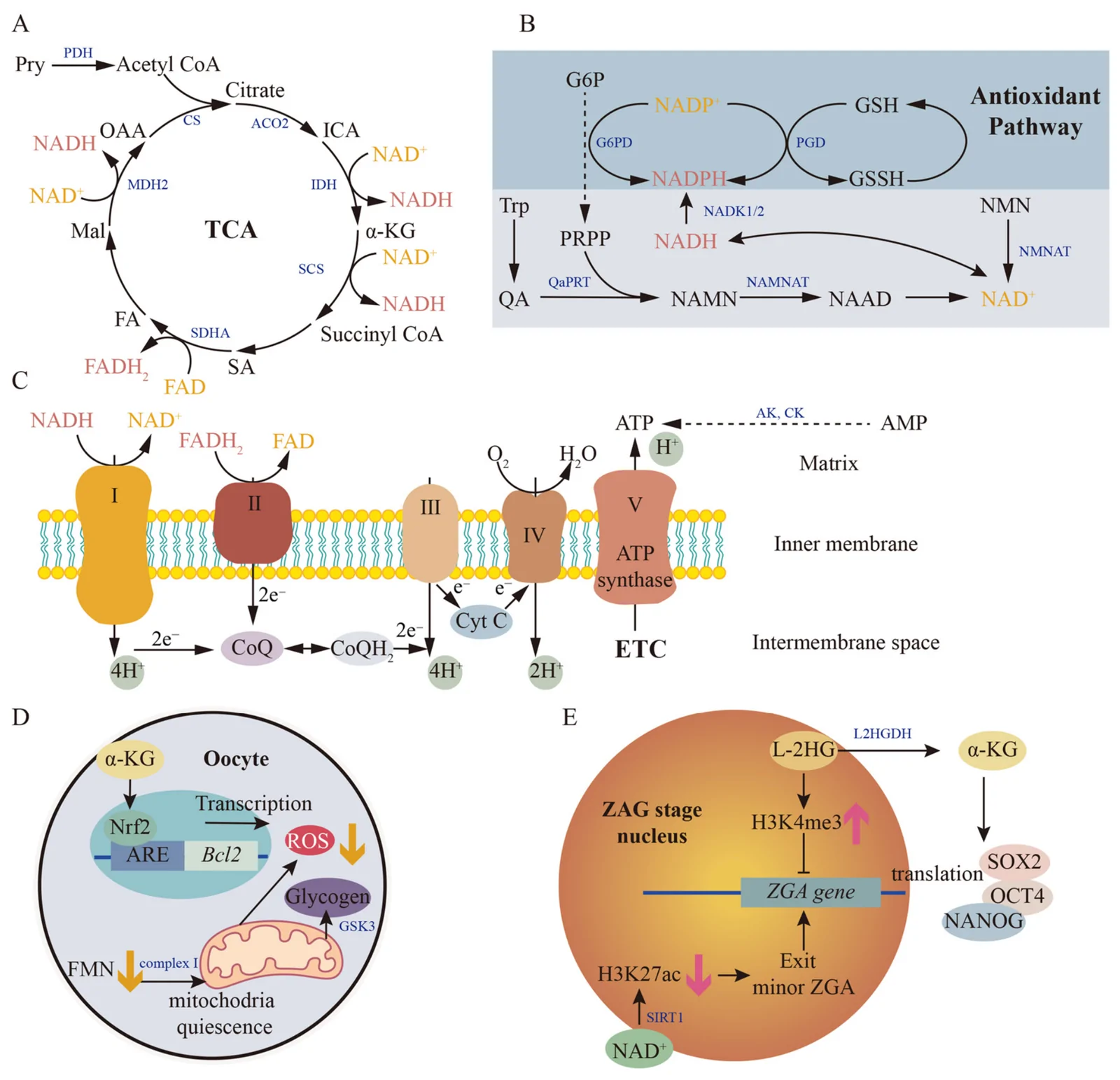
Important redox metabolism pathways during early embryonic development. (**A**) Tricarboxylic acid cycle. Pyr: pyruvic acid; PDH: pyruvate dehydrogenase; CS: citrate synthase; ACO2: aconitase 2; ICA: isocitrate acid; IDH: isocitrate dehydrogenase; α-KG: alpha-ketoglutarate; NAD^+^/NADH: nicotinamide adenine dinucleotides; SCS: succinyl-CoA synthetase; SA: succinic acid; SDHA: succinate dehydrogenase; FA: fumaric acid; FAD2^+^/FADH_2_: flavin adenine dinucleotide; Mal: malic acid; MDH2: malate dehydrogenase 2; OAA: oxaloacetate. (**B**) Antioxidant pathway. Trp: tryptophan; QA: quinolinic acid; G6P: glucose-6-phosphate; G6PD: glucose-6-phosphate dehydrogenase; PRPP: 5-Phosphoribosyl-1-pyrophosphate; QaPRT: quinolinic acid phospho-ribosyl-transferase; NAMN: nicotinic acid mononucleotide; NAMNAT: nicotinic acid mononucleotide adenylyl transferase; NAAD: nicotinic acid adenine dinucleotide; NMN: nicotinamide mononucleotide; NMNAT: nicotinamide mononucleotide adenylyl transferase; NADK1/2: NAD^+^ kinase 2; NADP/NADPH: nicotinamide adenine dinucleotide phosphate; GSH: glutathione; GSSG: glutathione disulfide. (**C**) ATP synthesis, CoQ/CoQH_2_: coenzyme Q; Cyt C: cytochrome C; AMP: adenosine monophosphate; ATP: adenosine triphosphate; AK: adenylate kinases; CK: creatine kinases. (**D**) Regulation of energetic metabolism in oocyte. Nrf2: Nuclear transcription factor E2-related factor 2; ARE: antioxidant response elements; Bcl2: B-cell lymphoma-2; ROS: reactive oxygen species; FMN: flavin mononucleotide; GSK3: glycogen synthase kinase 3. (**E**) Regulation of energetic metabolism during the ZGA stage. L-2HG: L-2-hydroxyglutarate; L2HGDH: L-2-hydroxyglutarate dehydrogenase; SOX2: SRY-box transcription factor 2; NANOG: Nanog homeobox.

Sharpley et al. followed the fate of metabolites from pyruvate catabolism using U13C-labelled pyruvate. The results confirmed that during the two-cell stage in mice, Asp is derived from oxaloacetate (OAA) and converted into malate via the Asp-Mal shuttle [101]. This process is coupled to NADH/NAD and may involve a change in the cellular redox state (Figure 5A and Figure 6A). Glutamate (Glu) is derived from α-KG [101], glutamine (Gln) is interconverted with Glu, and Gln has important effects in the differentiation of the three germ layers. Lu’s study showed that in hPSCs, the differentiation of endoderm and mesoderm requires the α-KG produced by Gln metabolism but not glucose or pyruvate and that ectodermal differentiation is dependent on the activation of mTORC1 by Gln for completion, suggesting an effect of Gln in embryonic cell fates [138] (Figure 5C). Metabolism of this specific source may be associated with related transaminases. In addition, decreased α-KG production from glutamate was associated with increased expression of Otx2 and its targets Dnmt3a/b, correlating with elevated DNA methylation [139]. This study mechanistically explains the link between amino acid metabolism and epigenomic modifications of the genome (Figure 5C). Glycine (Gly) levels are highest in the two-cell embryo due to the high expression of the amino acid transporter protein Slc6a9. Gly undergoes catabolism at ZGA and is significantly decreased in the blastocysts by conversion to purine nucleobase [101]. ASP involves the production of purines and pyrimidines.

#### 3.3.2. One-Carbon Metabolism

SGOC pathway products are elevated during meiotic resumption and then decrease in mature oocytes [121]. The metabolites taurine and hypotaurine were elevated, and tryptophan metabolite indole compounds were decreased. The direction of tryptophan metabolism is shown to inhibit the biosynthesis of indole compounds and promote the de novo synthesis of NAD^+^ (Figure 5A,B). It has been shown that there are significant effects of NAD^+^ in both mitochondrial functions and epigenetic modifications of embryonic development, as will be detailed in subsequent sections (Figure 6B,C). These results suggest that intense one-carbon metabolism occurs for oocyte maturation [96]. S-adenosylmethionine (SAM), an intermediate product of the methionine cycle, is a methyl group donor in many biochemical reactions (Figure 5A), and the elevation of SAM during oocyte maturation may be involved in the methylation of certain enzymes or the methylation of specific genes (e.g., imprinted genes).

### 3.4. Energetic Metabolism

Embryonic development is an energy-demanding process. However, mitochondria in early oocytes have unique characteristics. Studies of ATP dynamic changes in early oocytes have shown that ATP levels decline sharply after GVBD and that ATP consumption in MI and MII oocytes is higher than at the GV stage and peaks during polar body extrusion [140]. The major pathways involved in energetic and redox metabolism are summarized in Figure 6A–C.

#### 3.4.1. Tricarboxylic Acid Cycle (TCA)

Studies in which glucose destination was labeled with 13C isotopes confirmed low glucose uptake in oocytes and the near-derivation of acetyl-CoA from pyruvate and lactate, suggesting that early oocytes are not generating acetyl-CoA and the TCA by glucose but rather uptake of pyruvate from the environment [101], which is metabolically generated from CCs and is involved in the associated TCA [103].

Numerous reports in the literature have discussed the function of α-KG in promoting embryonic development, which may involve increasing mitochondrial activity and inhibiting apoptosis. α-KG activates the Nrf2/ARE signaling pathway to promote antioxidant targets against oxidative stress in IVM. Active Nrf2 may promote Bcl2 transcription and inhibits apoptosis [141] (Figure 6D).

As mentioned previously, α-KG mitigates the damage caused by pyruvate deficiency and allows embryos to develop to the blastocyst stage [103]. Follow-up studies confirmed that α-KG is derived from the catabolism of pyruvate rather than glucose [111], corroborating previous studies. Moreover, α-KG was not promoting embryonic development by regulating energy or redox balance, suggesting that another function of α-KG exists to promote further embryonic development.

In addition, α-KG increased the expression of porcine embryo pluripotency-related genes (e.g., OCT4, NANOG and SOX2). Recent studies have shown that 2-hydroxyglutarate (2-HG), produced by isocitrate dehydrogenase (IDH), is a competitive inhibitor of α-KG and inhibits the development of two-cell embryos into blastocysts by increasing the level of H3K4me3 methylation, which may contribute to a decrease in pluripotency genes (e.g., Nanog) [142]. The high concentration of 2-HG in two-cell embryos and the high level of α-KG in blastocysts are caused by the gradual increase in the level of TCA as the embryo develops and the gradual expression of L-2-hydroxyglutarate dehydrogenase (L2HGDH), which oxygenates L-2-HG to α-KG (Figure 6E).

#### 3.4.2. Electron Transport Chain (ETC)

Inhibiting mitochondrial oxidative phosphorylation in oocytes elicits a steep decline in intra-oocyte ATP, while inhibiting glycolysis does not affect ATP [140], demonstrating that mitochondria are the primary source of ATP in the oocyte. The mtDNA contains 13 proteins involved in the electron transport chain [143]. Its copy number gradually increases during folliculogenesis to oocyte maturation to form the electron transport chain [144] (Figure 6B). During the oogenesis to morula stage in human embryos, mitochondria present a unique rounded and hooded morphology with fewer cristae and less energy production efficiency in the electron transport chain than somatic mitochondria [55]. Sieber et al. found that mitochondria in mature Drosophila and Xenopus oocytes enter a low-activity ETC state by glycogen synthase kinase 3 (GSK3) to accumulate extensive glycogen for the developmental competence of the oocyte [145]. Recently, Rodríguez-Nuevo et al. combined live-cell imaging and proteomics to demonstrate the deletion of flavin mononucleotide (FMN) in human and early Xenopus oocytes, which results in incomplete assembly of mitochondria complex I and reduced ETC activity [146] (Figure 6E).

Mitochondria respiration provides electron acceptors that promote cell proliferation by supporting ASP biosynthesis [147]. Under normal conditions, oxidative glutamine metabolism is the main source of ASP in cells; when ETC is inhibited, reduced glutamine metabolism produces mainly ASP, dependent on GOT1 regulation [97]. This study suggests that Gln may depend on GOT1 to regulate ASP synthesis during embryonic development and thus plays a role in regulating cell proliferation.

#### 3.4.3. Nad^+^

NAD^+^ synthesis was enhanced for oocyte maturation [96]. Treatment with the NAD^+^ metabolic precursor nicotinamide mononucleotide (NMN) or overexpression of NAD-dependent deacetylase SIRT2 in mouse tissues confirms that NAD^+^ modulates SIRT2 activity and restores fertility by restoring oocytes in aged animals [110,148] (Figure 6B). The function of NAD^+^ may involve the regulation of spindle structure and chromosome alignment to avoid the euploidy of the genome after oocyte meiosis [110] and also the regulation of the distribution of cortical granules and ovastacin to ensure that sperm binding site zona pellucida glycoprotein 2 (ZP2) is intact before fertilization, thereby enhancing the fertilization of senescent oocytes [148]. In preimplantation embryos of mouse blastocyst stages, the metabolic cofactor NAD^+^ and the deacetylase SIRT1 remove zygotic H3K27ac in the late two-cell stage for the exit of minor ZGA [51] (Figure 6E).

#### 3.4.4. Oxidative Stress

There is usually a positive correlation between ATP levels in human oocytes for mitotic spindle formation at maturity, fertilization capacity after maturation, and blastocyst development after fertilization. However, Nagano [149] and Iwata et al. [150] reported that bovine oocytes with abnormally high ATP levels were positively associated with poorer developmental competence and maternal senescence in mature bovine oocytes. The phenomenon may be due to ROS, by-products of mitochondria metabolism, which is almost unavoidable during oocyte maturation.

Oxidative stress results from the imbalance between the production and elimination of ROS in the cell. During oocyte culture in vitro, the balance between producing and eliminating intracellular ROS is lost because of rapid temperature changes during oocyte collection and exposure to visible light. This directly affects the meiosis process, cytoskeletal structure, and gene expression, thus affecting oocyte quality. Low metabolic activity at early stages is thought to keep ROS levels low, hence avoiding DNA damage at a totipotent stage (Figure 6D). The dynamic changes in metabolic pathways and their products across different embryonic stages are summarized in Table 3.

## 4. Perspectives for the Optimization of Embryonic Development

### 4.1. Using Metabolism to Predict the Level of Embryonic Development

In mice and cattle, FAO levels increased during oocyte maturation [118,119,120,121] but decreased in pigs [96], suggesting that lipid metabolism is affected by many pathways, not only CPT but also TCA, and that species differences may cause these changes. Although most of the genes are conserved, there are still a few species-specific factors, which, although they cause some problems in our study, can also be used as specific expression factors to indicate the direction of metabolism. Therefore, continued research is needed to define species differences. Moreover, we highlight that while glycolytic suppression during early cleavage appears conserved across mice, humans, and cattle, lipid oxidation dynamics diverge markedly between species, likely reflecting differences in maternal lipid reserves and litter size.

### 4.2. Regulation of Metabolism Improves Embryonic Development

It was shown that mouse embryos in diapause have unique metabolic characteristics, including up-regulation of glycolysis, up-regulation of cholesterol synthesis and uptake [151]. Increasing AMPK to inhibit mTOR activity by starvation modeling or other methods (e.g., increased Lkb1 levels, high glutamine levels) reduces acetyl-CoA and H4K16Ac levels to maintain embryos in a stagnant state to prolong the time of embryo preservation.

Many metabolites are closely linked to epigenetic regulation. Some intermediates or end-products of cellular metabolic pathways directly modify DNA or histones, such as acetyl-CoA and lactate, while others regulate epigenetic modifications such as NAD^+^, VB1, α-KG. Numerous alterations in epigenetic modifications accompany the process of embryo development, and clarifying these metabolic pathways affecting epigenetic modifications is beneficial for improving the success of ART, blastocyst rate and embryo implantation.

Inhibition of TET1, an α-KG and Fe2^+^-dependent dioxygenase, leading to aberrant DNA methylation profiles, including incomplete demethylation of key regulators of meiosis and transposable elements, and ultimately causing reduced germ cell numbers, delayed meiosis and reduced fecundity in adulthood [152]. Recent mouse studies have shown that excessive glucose levels increase the methylation of the paternal pronucleus gene GCK by down-regulating TET3 in oocytes [153]. Furthermore, thiamine pyrophosphate (TPP), the active form of VB1 (thiamine), is a cofactor of PDH, the E1 catalytic component of the pyruvate dehydrogenase complex (PDC) in nutrient metabolism, and is required for acetyl-CoA synthesis and acetylation of core histones [154]. A high-fat diet (HFD) impairs maternal VB1 intake, which reduces the number of primordial follicles in the zygotic ovary by decreasing the metabolism of acetyl-CoA [155]. Regulation of TCA kinases may have a regulatory effect on histone acetylation and DNA methylation levels.

In addition, changes in the direction of metabolism may be one of the factors that closely correlate the metabolic state of cells with embryonic development. Nucleotide biosynthesis was reduced and TCA metabolites increased without Gln, whereas purine metabolism increased when GLS1 was inhibited, suggesting that modulation of GLS1 is different from the mere removal or supplementation of Gln but rather modulates the direction of metabolism to some extent [138]. During oocyte maturation, the ETC is in a prohibited state [146]. It may result from long periods of oocyte stasis to resist ROS damage. Therefore, the direction of ASP metabolism in oocytes at this time is biased towards the Gln reductive carboxylation pathway [97]. ASP is a precursor for purine and pyrimidine synthesis [156]; by studying GOT1, the enzyme that regulates the production of ASP from Gln, there may be an important influence on the abnormal development of oocytes.

### 4.3. Translational Implications for Embryo Culture and Assisted Reproduction

Beyond their mechanistic roles during early embryonic development, the metabolic pathways outlined above carry direct translational value for refining embryo culture systems and evaluating embryo quality in ART.

Accumulating evidence demonstrates that α-KG supplementation promotes meiotic maturation and blastocyst development in porcine oocytes by activating the Nrf2/ARE antioxidant pathway [141]. Meanwhile, NAD^+^ precursors such as NMN restore oocyte quality and fertility in aged mice by preserving sirtuin activity and ensuring proper spindle assembly [110,148]. Similarly, arginine supplementation improves oocyte quality via enhanced mitophagy [137]. Collectively, these findings reveal that supplementation of culture media with specific metabolites improves developmental outcomes, supporting formulation optimization of culture media for both human ART and livestock IVF.

Given the tight correlation between metabolic activity and embryonic developmental competence, metabolic parameters have been proposed as non-invasive biomarkers for embryo quality. Consistent with the glycolysis–trophectoderm fate relationship described above [111], real-time monitoring of lactate/pyruvate turnover and glycolytic flux in spent culture media can complement conventional morphological grading [157]. Likewise, recently developed live-cell lactate sensors such as FiLa enable detection of lactate-driven H3K18 acetylation during ZGA [104], highlighting the potential of real-time metabolic imaging to assess embryonic developmental stage and competence without invasive sampling.

As noted in Section 4.1, interspecies differences in lipid metabolism directly shape culture strategies for in vitro embryo production in livestock. For lipid-rich bovine and porcine embryos, modulation of lipolysis or antioxidant supplementation may be required to mitigate lipotoxicity. Furthermore, deeper insight into the metabolic requirements governing embryonic diapause via AMPK/mTOR signaling [151] may yield novel strategies to extend the pre-transfer storage lifespan of IVP embryos, with promising applications in genetic resource conservation and international livestock breeding programs. Taken together, these examples illustrate that metabolomic profiling and targeted metabolic interventions serve as powerful complements to morphology-based assessment and can substantially improve the efficacy and performance of assisted reproductive technologies.

Finally, it is important to note that specific proteins affect enzyme activity and function. For example, Wu’s study showed that Plin2 protects LDs from ATGL catabolism in the early stages of embryonic development [130]. This leads to differences in enzyme levels and metabolic processes, making our study difficult and contradictory. It is difficult to decipher the alterations in individual metabolites, as they may participate in several pathways. For a given metabolite, elevation in its concentration could represent increased production, decreased consumption, or both. So, we must consider changes during development in metabolic pathways based on the transcripts of genes encoding metabolic enzymes. In addition, metabolic activities are strongly influenced by allosteric regulation and post-translational modifications in crucial enzymes, and these modifications often play an essential role in regulating cell signal transduction, development, and other processes.

### 4.4. Metabolic Links to Infertility, Implantation Failure, and Recurrent Pregnancy Loss

Metabolic dysregulation is increasingly implicated in reproductive pathologies beyond the ART context discussed above. At the level of the follicle and oocyte, metabolomic profiling of follicular fluid has revealed altered glucose, lactate, pyruvate, amino acid, and lipid signatures in women with endometriosis, diminished ovarian reserve, and polycystic ovary syndrome, pointing to a link between systemic metabolic status and oocyte competence [158,159], consistent with the granulosa cell–oocyte metabolic coupling described in Section 2.1.2.

Implantation failure and recurrent pregnancy loss (RPL) similarly reflect disrupted metabolic communication at the maternal–embryo interface. Lipid-derived mediators contribute to endometrial receptivity [134], and multi-omics analyses of recurrent implantation failure (RIF) endometrium have identified concurrent abnormalities in amino acid, glycerophospholipid, and energy metabolism [160], extending the HBP/O-GlcNAcylation-dependent mechanisms discussed in Section 3.1.3. In RPL, endometrial fatty acid metabolism is disrupted during the implantation window [161], and hyperhomocysteinemia-associated RPL has been linked to impaired glutathione metabolism and ferroptotic signaling in endometrial stromal cells [162], paralleling the γ-glutamyl cycle and one-carbon metabolism pathways introduced in Section 3.3.2.

These associations point to metabolic profiling as a candidate source of biomarkers for infertility and implantation-related disorders, though most evidence to date derives from case–control metabolomic comparisons rather than mechanistic or interventional studies, and prospective validation in larger cohorts remains lacking.

## 5. Conclusions

Metabolism serves not merely as an energy supplier for early mammalian embryonic development but also as a dynamic regulatory hub that links nutrient availability, redox homeostasis, biosynthetic processes, cellular signaling, epigenetic modifications, and cell fate decisions. As development proceeds from oocyte maturation to the preimplantation stage, metabolic pathways and metabolites exhibit marked stage-specific alterations, which are tightly coupled with embryonic developmental competence. These metabolic signatures, however, vary substantially across species, developmental stages, tissue microenvironments and experimental setups; hence, results obtained from individual animal models require careful interpretation.

From a translational standpoint, embryonic metabolic profiling holds promise for optimizing embryo culture and IVP, developing non-invasive embryo evaluation strategies, and dissecting metabolic defects underlying infertility, implantation failure and recurrent pregnancy loss. Even so, most candidate metabolic biomarkers and intervention strategies are still confined to experimental or early translational phases. Upcoming research combining metabolomics with single-cell transcriptomics, epigenomics, proteomics, lipidomics and spatial omics technologies is critical to dissecting cell-resolved and stage-resolved metabolic states. Notably, putative metabolic pathways must be validated via functional perturbation, rescue assays, and prospective clinical or IVP investigations. These multi-omics integrative frameworks will help distinguish whether metabolic shifts act as causal drivers, secondary outcomes, or adaptive responses in early embryogenesis. Ultimately, such efforts will solidify the foundation for precision reproductive medicine and in vitro embryo production.

## Figures and Tables

**Table 1:** Factors affecting the early development of the embryo or oocyte.

Factor	Phase	Advantageous Phenotype	Reference
ATP level	GV oocyte	Glucose uptake by CCs	[27]
Chromatin structure	GV oocyte	Surrounded nucleolus type	[29,30]
Maturation of oocyte	GV oocyte	Maintaining oocyte meiotic arrest	[24]
	MI oocyte	Nucleus and cytoplasm synchronous maturation	[31]
Mitochondria	MI oocyte	Accumulate around the spindle	[32]
	Mature oocyte	Distributed in the cytoplasm large number	[33,34]
Chromatin assembly	Oocyte to blastocyst	Raise	[35]
DNA methylation	Oocyte, ZGA	Reduce	[36,37,38]
	Morula, blastocyst	Raise	[39,40,41,42,43]
H3K4me3	Human GV and MI oocyte	Raise	[44]
	Mouse MII oocyte	Reduce	[45]
	ZGA	Raise	[46]
H3K27me3	Oogenesis	Raise	[47]
	ZGA	Asymmetric loss in mouse, global loss in human	[44,48]
	Blastocyst	Loss in maternal genomes	[45]
H3K9me3	ZGA	Reduce	[49,50]
	Post-implantation embryos	Raise	[49]
H3K27ac	ZGA	Reduce	[51]
	Morula, Blastocyst	Raise	
Histone crotonylation	Blastocyst	Raise	[52,53]

**Table 2:** Pathways, kinases and products.

Categories	Pathway	Kinases	Products
Glucose Metabolism	Glycolysis	PKM2 LDH [92]	Pyr Lac
	PPP	G6PD	NADPH, PRPP
	HBP	OGT [93]	UDP-GlcNAc
Lipid Metabolism	FAO	CPT	Acetyl-CoA
	de novo synthesis	ACC FANS ACSL	Malonyl-CoA FAs TAG
	NEFA synthesis	SCD [94] ELOVL [95]	MUFAs PUFAs
	Cholesterol	HSD17-b2 [96]	Cholesterol Pregnenolone
	SCFA metabolism	ACADS, ACOX3	Crotonyl-CoA [52]
Amino Acid Metabolism	SGOC	SHMT	SAM
	Catabolism of Arg	AMD1, AZIN2 [96]	Spermidine
	Asp synthesis	GOT1 [97]	Asp
	Pro synthesis	PYCR1/2 [98]	Pro
	Taurine synthesis	CDO1 [96]	Taurine
	γ-Glutamyl cycle	GCLC [92]	GSH
Energetic Metabolism	TCA	IDH IDH, SCS, MDH2	α-KG NADH
	NAD^+^ synthesis [99]	NAMNAT NMNAT	NAD^+^
	Coupling	LDH PYCR1/2 [98]	
	Adenosine salvage	AK, CK	ATP
	OXPHOS	ETC	

**Table 3:** Changes in metabolic pathways and products under different embryonic developmental processes.

Developmental Stages	Metabolites OR Kinases	Direction	Pathway	Species and Reference
Early oocytes	NADH	Reduce	ETC	Drosophila, Xenopus and human [145,146]
	NADPH	Enhance	PPP	Xenopus [108]
Maturing oocyte	NADPH	Enhance	PPP	Porcine [96,121]
	Citrate	Enhance	TCA	Porcine and mouse [96,121]
	PUFA	Reduce	Lipid synthesis	Mouse [121]
	bile acids	Reduce	Bile acids synthetics	Porcine [96]
	FA	Reduce	FAO	Mouse, cattle [96,114,115,116,117,118]
	Spermidine	Enhance	Catabolism of Arg and Pro	Porcine [96]
	SAM	Enhance	SGOC	Porcine, mouse [96,121]
	NAD^+^	Enhance	NAD^+^ synthesis; catabolism of Trp	Porcine [96], mouse [110,148]
	PRPP	Enhance	Nucleotides synthetics	Porcine [96]
	IMP	Enhance	Nucleotides synthetics	Mouse [121]
	UDP-GlcNAc	Enhance	HBP	Mouse [112]
	α-KG	Enhance	TCA	Porcine [141]
ZGA	PFK	Reduce	Glycolysis	Mouse [102]
	Lac	Enhance	Glycolysis	Mouse and human [104]
	Gly	Enhance	Catabolism of Gly	[101]
Morula	LDs	Enhance	Lipid synthesis	Cattle [95,126] and Mouse [129]
Blastocyst	Acetyl-CoA	Enhance	FAO	hESCs [124]
	LDs	Enhance	Lipid de novo synthesis	ESCs [130], TE [129]
	MYC	Enhance	EMP	hESCs [107]
	Carnitine	Enhance	FAO	mESCs [125]
	SCD1, PUFA	Enhance	Phospholipid metabolism	Mouse [94,122] and cattle [123]
	UDP-GlcNAc	Enhance	HBP	Mouse [111]
	α-KG	Enhance	TCA	Mouse [111,142] and porcine [141]

## Data Availability

No new data were created or analyzed in this study.

## Abbreviations

2-HG: 2-hydroxyglutarate; 5hmC: 5-hydroxymethylcytosine; 5mC: 5-methylcytosine; 5m-THF: 5-methyl tetrahydrofolate; α-KG: alpha-ketoglutarate; γ-Glu-Cys: gamma-glutamyl–cysteine; ACC: acetyl-CoA carboxylase; ACADS: acyl-CoA dehydrogenase short chain; ACO2: aconitase 2; ACOX3: Acyl-CoA oxidase 3; ACSL: long-chain acyl-CoA synthetase; ADAMTST1: ADAM metallopeptidase with thrombospondin type 1 motif 1; AG: androgenotes; AK: adenylate kinases; AMD1: adenosylmethionine decarboxylase 1; AMP: adenosine monophosphate; AMPK: AMP-activated protein kinase; aPKC: atypical protein kinase C; ARE: antioxidant response elements; Arg: arginine; ART: assisted reproductive technology; Asp: aspartate; ATAC-seq: assay for transposase-accessible chromatin with sequencing; ATGL: adipose triglyceride lipase; ATP: adenosine triphosphate; AZIN2: antizyme inhibitor 2; Bcl2: B-cell lymphoma 2; BMP-15: bone morphogenetic protein 15; BTG4: BTG anti-proliferation factor 4; C3a: complement component 3a; CaMKII: calcium/calmodulin-dependent protein kinase II; cAMP: cyclic adenosine monophosphate; CAF-1: chromatin assembly factor 1; CAMs: cell adhesion molecules; CCL4: C-C motif chemokine ligand 4; CCs: cumulus cells; CCR5: C-C motif chemokine receptor 5; CDK1: cyclin-dependent kinase 1; CDO1: cysteine dioxygenase 1; CDX2: caudal type homeobox 2; cGMP: cyclic guanosine monophosphate; CHAF1a: chromatin assembly factor 1 subunit a; CHAF1b: chromatin assembly factor 1 subunit b; CH3: methyl; ChIP-seq: chromatin immunoprecipitation sequencing; CIDEA: cell death-inducing DFFA-like effector A; CK: creatine kinases; CLs: cardiolipins; CMA: chaperone-mediated autophagy; CoA: coenzyme A; COCs: cumulus-oocyte complexes; CoQ/CoQH2: coenzyme Q; CoX: cyclooxygenase; CPT: carnitine palmitoyl-transferase; CS: citrate synthase; Cyt C: cytochrome C; CUT&RUN: cleavage under targets and release using nuclease; CUT&Tag: cleavage under targets and tagmentation; CX43: connexin 43; CXCL8: C-X-C motif chemokine ligand 8; CXCR2: C-X-C motif chemokine receptor 2; Cys: cysteine; DAG: diacylglycerol; DC: dendritic cells; DNA: deoxyribonucleic acid; DNase-seq: DNase I hypersensitive sites sequencing; DNMT: DNA methyltransferase; DNMT1: DNA methyltransferase 1; DNMT3a/b: DNA methyltransferase 3 alpha/beta; DNMT3B: DNA methyltransferase 3 beta; E2: estrogen; ECM: extracellular matrix; ED: embryonic diapause; EGA: embryonic genome activation; EGFR: epidermal growth factor receptor; ELOVL: elongases of very long-chain fatty acids; EMP: Embden–Meyerhof–Parna pathway; EPI: epiblast; Ep300: E1A binding protein p300; EREG: epiregulin; ESCs: embryonic stem cells; ETC: electron transport chain; EVs: extracellular vesicles; FAD2+/FADH2: flavin adenine dinucleotide; FAs: fatty acids; FAO: fatty acid beta-oxidation; FGF-8: fibroblast growth factor 8; FiLa: fluorescent lactate sensor; FMN: flavin mononucleotide; FN1: fibronectin 1; FOXO1: Forkhead box O1; FSH: follicle-stimulating hormone; G: glucose; G3P: 3-phosphoglycerate; G6P: glucose-6-phosphate; G6PD: glucose-6-phosphate dehydrogenase; GATA3: GATA binding protein 3; GCK: glucokinase; GCLC: glutamate–cysteine ligase catalytic subunit; GCs: granulosa cells; GDL: gluconolactone; GFAT: fructose-6-phosphate aminotransferase; GlcN-6p: glucosamine-6-phosphate; Gln: glutamine; Glu: glutamate; GLUT1: glucose transporter 1; GLUT4: glucose transporter 4; Gly: glycine; GNPDA: glucosamine-6-phosphate deaminase; GOT1: glutamate oxaloacetate transaminase 1; GPi: glucose-6-phosphate isomerase; GS: glutamine synthetase; GSH: reduced glutathione; GSK3: glycogen synthase kinase 3; GSS: glutathione synthetase; GSSG: oxidized glutathione; GV: germinal vesicle; GVBD: germinal vesicle breakdown; H: histone; H2O2: hydrogen peroxide; H3.1/3.2: histone H3.1/3.2; H3.3: histone H3.3; H3K4me2/3: histone H3 lysine 4 di/trimethylation; H3K4me3: histone H3 lysine 4 trimethylation; H3K9me2/3: histone H3 lysine 9 di/trimethylation; H3K9me3: histone H3 lysine 9 trimethylation; H3K27me2/3: histone H3 lysine 27 di/trimethylation; H3K27me3: histone H3 lysine 27 trimethylation; H3K27ac: histone H3 lysine 27 acetylation; H3K18cr: histone H3 lysine 18 crotonylation; H3K9cr: histone H3 lysine 9 crotonylation; H3K23cr: histone H3 lysine 23 crotonylation; H3K36cr: histone H3 lysine 36 crotonylation; H3K27cr: histone H3 lysine 27 crotonylation; H3K14cr: histone H3 lysine 14 crotonylation; H3K18la: histone H3 lysine 18 lactylation; H4K16Ac: histone H4 lysine 16 acetylation; HA: hyaluronic acid; HAT: histone acetyltransferase; HBP: hexosamine biosynthesis pathway; Hcy: homocysteine; HDAC: histone deacetylase; HDM: histone demethylase; HFD: high-fat diet; HK: hexokinase; HMG-CoA: 3-hydroxy-3-methylglutaryl-CoA; HMGCR: 3-hydroxy-3-methylglutaryl-CoA reductase; HMT: histone methyltransferase; HPLC-MS: high-performance liquid chromatography–mass spectrometry; Hsc70: heat shock cognate 70; HSP90: heat shock protein 90; HSL: hormone-sensitive lipase; ICA: isocitrate acid; ICM: inner cell mass; ICRs: imprinting control regions; IDH: isocitrate dehydrogenase; IMP: inosine monophosphate; IVF: in vitro fertilization; IVM: in vitro maturation; IVP: in vitro production; Kat6a: lysine acetyltransferase 6A; KLF17: Krueppel-like transcription factor 17; L-2HG: L-2-hydroxyglutarate; L2HGDH: L-2-hydroxyglutarate dehydrogenase; Lac: lactic acid; LC3: microtubule-associated protein 1A/1B light chain 3; LCFAs: long-chain fatty acids; LC-MS/MS: liquid chromatography–tandem mass spectrometry; LDH: lactate dehydrogenase; LDs: lipid droplets; LH: luteinizing hormone; Lkb1: liver kinase B1; LPA: lysophosphatidic acid; LTR: long terminal repeat; LV: lysosome vesicles; M: macrophage; MAG: monoacylglycerol; MAGL: monoacylglycerol lipase; Mal: malic acid; MCT: monocarboxylate transporter; MGCs: mural granulosa cells; MDH2: malate dehydrogenase 2; Met: methionine; METTL3: methyltransferase-like 3; METTL14: methyltransferase-like 14; MGCs: mural granulosa cells; MI: metaphase I; MII: metaphase II; MPF: maturation-promoting factor; m6A: N6-methyladenosine; mESCs: mouse embryonic stem cells; mRNA: messenger RNA; mtDNA: mitochondrial DNA; mTOR: mammalian target of rapamycin; mTORC1: mTOR complex 1; MUFAs: monounsaturated fatty acids; MVA: mevalonate; MZT: maternal-to-zygotic transition; N: neutrophil; NAD+: nicotinamide adenine dinucleotide (oxidized); NADH: reduced nicotinamide adenine dinucleotide; NADK1/2: NAD+ kinase 1/2; NADP+: nicotinamide adenine dinucleotide phosphate; NADPH: reduced nicotinamide adenine dinucleotide phosphate; NAAD: nicotinic acid adenine dinucleotide; NAMN: nicotinic acid mononucleotide; NAMNAT: nicotinic acid mononucleotide adenylyl transferase; NANOG: Nanog homeobox; ncH3K4me3: non-canonical histone H3 lysine 4 trimethylation; NEFA: non-esterified fatty acid; NFYA: nuclear transcription factor Y subunit alpha; Nkap: NF-kappa-B-activating protein; NMN: nicotinamide mononucleotide; NMNAT: nicotinamide mononucleotide adenylyl transferase; NO: nitric oxide; NOMe-seq: nucleosome occupancy and methylome sequencing; Nrf2: nuclear transcription factor E2-related factor 2; NSN: non-surrounded nucleolus; OAA: oxaloacetate; OBOX: PRD-like homeobox domain transcription factor; OCT4: octamer-binding transcription factor 4; O-GlcNAc: O-linked N-acetylglucosamine; OGT: O-linked N-acetylglucosamine transferase; OXPHOS: oxidative phosphorylation; P4: progesterone; p48: CAF-1 subunit p48; PA: parthenogenetic activation; PB1: first polar body; PB2: second polar body; PC: phosphatidylcholine; PDC-E1: pyruvate dehydrogenase complex E1; PDH: pyruvate dehydrogenase; PE: phosphatidylethanolamine or primitive endoderm (context-dependent); PFK: phosphofructokinase; PG: prostaglandin or parthenogenotes (context-dependent); PGLS: 6-phosphogluconolactonase; PI: phosphatidylinositol; Plin2: perilipin 2; PLs: phospholipids; PN: pronuclei; Pol II: RNA polymerase II; PPP: pentose phosphate pathway; Pramel15: PRAME family member 15; Pro: proline; PRPP: 5-phosphoribosyl-1-pyrophosphate; PSC: pluripotent stem cell; PUFAs: polyunsaturated fatty acids; Pyr: pyruvic acid; PYCR1/2: pyrroline-5-carboxylate reductase 1/2; QA: quinolinic acid; QaPRT: quinolinic acid phosphoribosyl transferase; R5P: ribose 5-phosphate; RHOA: Ras homolog family member A; RNA: ribonucleic acid; ROS: reactive oxygen species; SA: succinic acid; SAH: S-adenosylhomocysteine; SAM: S-adenosylmethionine; SCD: stearoyl-CoA desaturase; SCD1: stearoyl-CoA desaturase 1; SCFA: short-chain fatty acid; scm6A-seq: single-cell m6A sequencing; SCS: succinyl-CoA synthetase; SDHA: succinate dehydrogenase; Ser: serine; SF3B1: splicing factor 3b subunit 1; SFAs: saturated fatty acids; SGOC: serine–glycine–one-carbon; SHMT: serine hydroxy methyltransferase; SIRT1: sirtuin 1; SIRT2: sirtuin 2; SLC2A4: solute carrier family 2 member 4; SLC6A9: solute carrier family 6 member 9; SMAD3: SMAD family member 3; SMs: sphingomyelins; SN: surrounded nucleolus; SOX2: SRY-box transcription factor 2; SP1: Sp1 transcription factor; S1P: sphingosine-1-phosphate; Stacc-seq: small-scale Tn5-assisted chromatin cleavage with sequencing; STAR: steroidogenic acute regulatory protein; T: T cells; TAG: triacylglycerol; TCA: tricarboxylic acid cycle; TCF3: transcription factor 3; TCF12: transcription factor 12; TDP-43: tar DNA binding protein 43; TE: trophectoderm; TEAD4: TEA-domain family member 4; TET: ten-eleven translocation; TET1: ten-eleven translocation 1; TET3: ten-eleven translocation 3; TF: transcription factor; TFAP2C: transcription factor AP-2 gamma; THF: tetrahydrofolate; TPP: thiamine pyrophosphate; Trp: tryptophan; tsRNA: tRNA-derived small RNA; TSP: trans-sulfuration pathway; TSS: transcription start site; TZPs: transzonal projections; UDP-GlcNAc: uridine diphosphate N-acetylglucosamine; UTP: uridine triphosphate; VB1: vitamin B1; VCAN: Versican; WTAP: WT1-associated protein; XCI: X chromosome inactivation; Xist: X-inactive specific transcript; YAP1: yes-associated protein 1; ZGA: zygotic gene activation; ZP2: zona pellucida glycoprotein 2; ZSA: zygotic splicing activation.

## References

[1] Kerr J.B., Myers M., Anderson R.A. (2013). The dynamics of the primordial follicle reserve. Reproduction.

[2] Westergaard C.G., Byskov A.G., Andersen C.Y. (2007). Morphometric characteristics of the primordial to primary follicle transition in the human ovary in relation to age. Hum. Reprod..

[3] Silva R.C., Báo S.N., Jivago J.L.P., Lucci C.M. (2011). Ultrastructural characterization of porcine oocytes and adjacent follicular cells during follicle development: Lipid component evolution. Theriogenology.

[4] Uyar A., Torrealday S., Seli E. (2013). Cumulus and granulosa cell markers of oocyte and embryo quality. Fertil. Steril..

[5] Da Silva-Buttkus P., Jayasooriya G.S., Mora J.M., Mobberley M., Ryder T.A., Baithun M., Stark J., Franks S., Hardy K. (2008). Effect of cell shape and packing density on granulosa cell proliferation and formation of multiple layers during early follicle development in the ovary. J. Cell Sci..

[6] Diaz F., O’BRien M., Wigglesworth K., Eppig J. (2006). The preantral granulosa cell to cumulus cell transition in the mouse ovary: Development of competence to undergo expansion. Dev. Biol..

[7] Bhardwaj J.K., Paliwal A., Saraf P., Sachdeva S.N. (2021). Role of autophagy in follicular development and maintenance of primordial follicular pool in the ovary. J. Cell. Physiol..

[8] Baerwald A.R., Adams G.P., Pierson R.A. (2011). Ovarian antral folliculogenesis during the human menstrual cycle: A review. Hum. Reprod. Update.

[9] Song Y., Zhang N., Zhang Y., Wang J., Lv Q., Zhang J. (2024). Single-Cell Transcriptome Analysis Reveals Development-Specific Networks at Distinct Synchronized Antral Follicle Sizes in Sheep Oocytes. Int. J. Mol. Sci..

[10] Liu Y.-X., Zhang Y., Li Y.-Y., Liu X.-M., Wang X.-X., Zhang C.-L., Hao C.-F., Deng S.-L. (2019). Regulation of follicular development and differentiation by intra-ovarian factors and endocrine hormones. Front. Biosci..

[11] Sharma A., Tiwari M., Gupta A., Pandey A.N., Yadav P.K., Chaube S.K. (2018). Journey of oocyte from metaphase-I to metaphase-II stage in mammals. J. Cell. Physiol..

[12] Sanders J.R., Jones K.T. (2018). Regulation of the meiotic divisions of mammalian oocytes and eggs. Biochem. Soc. Trans..

[13] Ikawa M., Inoue N., Benham A.M., Okabe M. (2010). Fertilization: A sperm’s journey to and interaction with the oocyte. J. Clin. Investig..

[14] Duan X., Sun S.-C. (2019). Actin cytoskeleton dynamics in mammalian oocyte meiosis. Biol. Reprod..

[15] Zhao C., Zhang N., Zhang Y., Tuersunjiang N., Gao S., Liu W., Zhang Y. (2020). A DNA methylation state transition model reveals the programmed epigenetic heterogeneity in human pre-implantation embryos. Genome Biol..

[16] Rulands S., Lee H.J., Clark S.J., Angermueller C., Smallwood S.A., Krueger F., Mohammed H., Dean W., Nichols J., Rugg-Gunn P. (2018). Genome-Scale Oscillations in DNA Methylation during Exit from Pluripotency. Cell Syst..

[17] Ju L.-F., Xu H.-J., Yang Y.-G., Yang Y. (2023). Omics Views of Mechanisms for Cell Fate Determination in Early Mammalian Development. Genom. Proteom. Bioinform..

[18] Wörheide M.A., Krumsiek J., Kastenmüller G., Arnold M. (2021). Multi-omics integration in biomedical research – A metabolomics-centric review. Anal. Chim. Acta.

[19] Wu H., Zhu R., Zheng B., Liao G., Wang F., Ding J., Li H., Li M. (2022). Single-Cell Sequencing Reveals an Intrinsic Heterogeneity of the Preovulatory Follicular Microenvironment. Biomolecules.

[20] Yang Y., Zhao C., Chen B., Yu X., Zhou Y., Ni D., Zhang X., Zhang J., Ling X., Zhang Z. (2023). Follicular fluid C3a-peptide promotes oocyte maturation through F-actin aggregation. BMC Biol..

[21] Sutton-McDowall M.L., Gilchrist R.B., Thompson J.G. (2010). The pivotal role of glucose metabolism in determining oocyte developmental competence. Reproduction.

[22] Zhang C.-H., Liu X.-Y., Wang J. (2023). Essential Role of Granulosa Cell Glucose and Lipid Metabolism on Oocytes and the Potential Metabolic Imbalance in Polycystic Ovary Syndrome. Int. J. Mol. Sci..

[23] El-Hayek S., Yang Q., Abbassi L., FitzHarris G., Clarke H.J. (2018). Mammalian Oocytes Locally Remodel Follicular Architecture to Provide the Foundation for Germline-Soma Communication. Curr. Biol..

[24] Winterhager E., Kidder G.M. (2015). Gap junction connexins in female reproductive organs: Implications for women’s reproductive health. Hum. Reprod. Updat..

[25] Sugiura K., Su Y.-Q., Diaz F.J., Pangas S.A., Sharma S., Wigglesworth K., O’Brien M.J., Matzuk M.M., Shimasaki S., Eppig J.J. (2007). Oocyte-derived BMP15 and FGFs cooperate to promote glycolysis in cumulus cells. Development.

[26] Sugiura K., Pendola F.L., Eppig J.J. (2005). Oocyte control of metabolic cooperativity between oocytes and companion granulosa cells: Energy metabolism. Dev. Biol..

[27] Wang Q., Chi M.M., Moley K.H. (2012). Live Imaging Reveals the Link Between Decreased Glucose Uptake in Ovarian Cumulus Cells and Impaired Oocyte Quality in Female Diabetic Mice. Endocrinology.

[28] Wen J., Wang G.-L., Yuan H.-J., Zhang J., Xie H.-L., Gong S., Han X., Tan J.-H. (2020). Effects of glucose metabolism pathways on nuclear and cytoplasmic maturation of pig oocytes. Sci. Rep..

[29] Lee J.B., Lee M.G., Lin T., Shin H.Y., Lee J.E., Kang J.W., Jin D.-I. (2019). Effect of oocyte chromatin status in porcine follicles on the embryo development in vitro. Asian-Australas. J. Anim. Sci..

[30] Ma J.-Y., Li M., Luo Y.-B., Song S., Tian D., Yang J., Zhang B., Hou Y., Schatten H., Liu Z. (2013). Maternal factors required for oocyte developmental competence in mice: Transcriptome analysis of non-surrounded nucleolus (NSN) and surrounded nucleolus (SN) oocytes. Cell Cycle.

[31] Ferrer-Vaquer A., Barragán M., Rodríguez A., Vassena R. (2019). Altered cytoplasmic maturation in rescued in vitro matured oocytes. Hum. Reprod..

[32] Dalton C.M., Carroll J. (2013). Biased inheritance of mitochondria during asymmetric cell division in the mouse oocyte. J. Cell Sci..

[33] Reader K.L., Stanton J.-A.L., Juengel J.L. (2017). The Role of Oocyte Organelles in Determining Developmental Competence. Biology.

[34] Van Blerkom J., Davis P., Alexander S. (2000). Differential mitochondrial distribution in human pronuclear embryos leads to disproportionate inheritance between blastomeres: Relationship to microtubular organization, ATP content and competence. Hum. Reprod..

[35] Wilkinson A.L., Zorzan I., Rugg-Gunn P.J. (2023). Epigenetic regulation of early human embryo development. Cell Stem Cell.

[36] Xu Q., Xie W. (2018). Epigenome in Early Mammalian Development: Inheritance, Reprogramming and Establishment. Trends Cell Biol..

[37] Giaccari C., Cecere F., Argenziano L., Pagano A., Galvao A., Acampora D., Rossi G., Mele B.H., Acurzio B., Coonrod S. (2024). A maternal-effect *Padi6* variant causes nuclear and cytoplasmic abnormalities in oocytes, as well as failure of epigenetic reprogramming and zygotic genome activation in embryos. Genes Dev..

[38] Tan J., Li Y., Li X., Zhu X., Liu L., Huang H., Wei J., Wang H., Tian Y., Wang Z. (2024). Pramel15 facilitates zygotic nuclear DNMT1 degradation and DNA demethylation. Nat. Commun..

[39] Schulz M., Teissandier A., Santaella E.D.L.M., Armand M., Iranzo J., El Marjou F., Gestraud P., Walter M., Kinston S., Göttgens B. (2024). DNA methylation restricts coordinated germline and neural fates in embryonic stem cell differentiation. Nat. Struct. Mol. Biol..

[40] Zhang Y., Xiang Y., Yin Q., Du Z., Peng X., Wang Q., Fidalgo M., Xia W., Li Y., Zhao Z.-A. (2018). Dynamic epigenomic landscapes during early lineage specification in mouse embryos. Nat. Genet..

[41] Dahlet T., Lleida A.A., Al Adhami H., Dumas M., Bender A., Ngondo R.P., Tanguy M., Vallet J., Auclair G., Bardet A.F. (2020). Genome-wide analysis in the mouse embryo reveals the importance of DNA methylation for transcription integrity. Nat. Commun..

[42] Andergassen D., Smith Z.D., Kretzmer H., Rinn J.L., Meissner A. (2021). Diverse epigenetic mechanisms maintain parental imprints within the embryonic and extraembryonic lineages. Dev. Cell.

[43] Andrews S., Krueger C., Mellado-Lopez M., Hemberger M., Dean W., Perez-Garcia V., Hanna C.W. (2023). Mechanisms and function of de novo DNA methylation in placental development reveals an essential role for DNMT3B. Nat. Commun..

[44] Xia W., Xu J., Yu G., Yao G., Xu K., Ma X., Zhang N., Liu B., Li T., Lin Z. (2019). Resetting histone modifications during human parental-to-zygotic transition. Science.

[45] Zhang B., Zheng H., Huang B., Li W., Xiang Y., Peng X., Ming J., Wu X., Zhang Y., Xu Q. (2016). Allelic reprogramming of the histone modification H3K4me3 in early mammalian development. Nature.

[46] Dahl J.A., Jung I., Aanes H., Greggains G.D., Manaf A., Lerdrup M., Li G., Kuan S., Li B., Lee A.Y. (2016). Broad histone H3K4me3 domains in mouse oocytes modulate maternal-to-zygotic transition. Nature.

[47] Yang X., Hu B., Hou Y., Qiao Y., Wang R., Chen Y., Qian Y., Feng S., Chen J., Liu C. (2018). Silencing of developmental genes by H3K27me3 and DNA methylation reflects the discrepant plasticity of embryonic and extraembryonic lineages. Cell Res..

[48] Inoue A., Jiang L., Lu F., Suzuki T., Zhang Y. (2017). Maternal H3K27me3 controls DNA methylation-independent imprinting. Nature.

[49] Wang C., Liu X., Gao Y., Yang L., Li C., Liu W., Chen C., Kou X., Zhao Y., Chen J. (2018). Reprogramming of H3K9me3-dependent heterochromatin during mammalian embryo development. Nat. Cell Biol..

[50] Matoba S., Liu Y., Lu F., Iwabuchi K.A., Shen L., Inoue A., Zhang Y. (2014). Embryonic Development following Somatic Cell Nuclear Transfer Impeded by Persisting Histone Methylation. Cell.

[51] Li J., Zhang J., Hou W., Yang X., Liu X., Zhang Y., Gao M., Zong M., Dong Z., Liu Z. (2022). Metabolic control of histone acetylation for precise and timely regulation of minor ZGA in early mammalian embryos. Cell Discov..

[52] Fang Y., Xu X., Ding J., Yang L., Doan M.T., Karmaus P.W., Snyder N.W., Zhao Y., Li J.-L., Li X. (2021). Histone crotonylation promotes mesoendodermal commitment of human embryonic stem cells. Cell Stem Cell.

[53] Gao D., Li C., Liu S.-Y., Xu T.-T., Lin X.-T., Tan Y.-P., Gao F.-M., Yi L.-T., Zhang J.V., Ma J.-Y. (2024). P300 regulates histone crotonylation and preimplantation embryo development. Nat. Commun..

[54] Tan J.-H., Wang H.-L., Sun X.-S., Liu Y., Sui H.-S., Zhang J. (2009). Chromatin configurations in the germinal vesicle of mammalian oocytes. Mol. Hum. Reprod..

[55] Bentov Y., Yavorska T., Esfandiari N., Jurisicova A., Casper R.F. (2011). The contribution of mitochondrial function to reproductive aging. J. Assist. Reprod. Genet..

[56] Lin M.T., Beal M.F. (2006). Mitochondrial dysfunction and oxidative stress in neurodegenerative diseases. Nature.

[57] Takeuchi T., Neri Q.V., Katagiri Y., Rosenwaks Z., Palermo G.D. (2005). Effect of Treating Induced Mitochondrial Damage on Embryonic Development and Epigenesis. Biol. Reprod..

[58] Chen Q., Yan M., Cao Z., Li X., Zhang Y., Shi J., Feng G.-H., Peng H., Zhang X., Zhang Y. (2016). Sperm tsRNAs contribute to intergenerational inheritance of an acquired metabolic disorder. Science.

[59] Cui L., Fang L., Zhuang L., Shi B., Lin C.-P., Ye Y. (2023). Sperm-borne microRNA-34c regulates maternal mRNA degradation and preimplantation embryonic development in mice. Reprod. Biol. Endocrinol..

[60] Vendrell X., de Castro P., Escrich L., Grau N., Gonzalez-Martin R., Quiñonero A., Escribá M.J., Domínguez F. (2024). Longitudinal profiling of human androgenotes through single-cell analysis unveils paternal gene expression dynamics in early embryo development. Hum. Reprod..

[61] Yuan S., Zhan J., Zhang J., Liu Z., Hou Z., Zhang C., Yi L., Gao L., Zhao H., Chen Z.-J. (2023). Human zygotic genome activation is initiated from paternal genome. Cell Discov..

[62] Liu B., Xu Q., Wang Q., Feng S., Lai F., Wang P., Zheng F., Xiang Y., Wu J., Nie J. (2020). The landscape of RNA Pol II binding reveals a stepwise transition during ZGA. Nature.

[63] Hu Y., Wang Y., He Y., Ye M., Yuan J., Ren C., Wang X., Wang S., Guo Y., Cao Q. (2024). Maternal KLF17 controls zygotic genome activation by acting as a messenger for RNA Pol II recruitment in mouse embryos. Dev. Cell.

[64] Ji S., Chen F., Stein P., Wang J., Zhou Z., Wang L., Zhao Q., Lin Z., Liu B., Xu K. (2023). OBOX regulates mouse zygotic genome activation and early development. Nature.

[65] Nie X., Xu Q., Xu C., Chen F., Wang Q., Qin D., Wang R., Gao Z., Lu X., Yang X. (2023). Maternal TDP-43 interacts with RNA Pol II and regulates zygotic genome activation. Nat. Commun..

[66] Tian Q., Yin Y., Tian Y., Wang Y., Wang Y., Fukunaga R., Fujii T., Liao A., Li L., Zhang W. (2024). Chromatin Modifier EP400 Regulates Oocyte Quality and Zygotic Genome Activation in Mice. Adv. Sci..

[67] Liu B., He Y., Wu X., Lin Z., Ma J., Qiu Y., Xiang Y., Kong F., Lai F., Pal M. (2024). Mapping putative enhancers in mouse oocytes and early embryos reveals TCF3/12 as key folliculogenesis regulators. Nat. Cell Biol..

[68] Gerri C., McCarthy A., Alanis-Lobato G., Demtschenko A., Bruneau A., Loubersac S., Fogarty N.M.E., Hampshire D., Elder K., Snell P. (2020). Initiation of a conserved trophectoderm program in human, cow and mouse embryos. Nature.

[69] Zhu M., Meglicki M., Lamba A., Wang P., Royer C., Turner K., Jauhar M.A., Jones C., Child T., Coward K. (2024). Tead4 and Tfap2c generate bipotency and a bistable switch in totipotent embryos to promote robust lineage diversification. Nat. Struct. Mol. Biol..

[70] Zhang Y., Yang Y., Qiao P., Wang X., Yu R., Sun H., Xing X., Zhang Y., Su J. (2022). CHAF1b, chromatin assembly factor-1 subunit b, is essential for mouse preimplantation embryos. Int. J. Biol. Macromol..

[71] SanMiguel J.M., Bartolomei M. (2018). DNA methylation dynamics of genomic imprinting in mouse development. Biol. Reprod..

[72] Inoue A., Zhang Y. (2014). Nucleosome assembly is required for nuclear pore complex assembly in mouse zygotes. Nat. Struct. Mol. Biol..

[73] Yan R., Cheng X., Gu C., Xu Y., Long X., Zhai J., Sun F., Qian J., Du Y., Wang H. (2023). Dynamics of DNA hydroxymethylation and methylation during mouse embryonic and germline development. Nat. Genet..

[74] Shen Y., Wei W., Zhou D.-X. (2015). Histone Acetylation Enzymes Coordinate Metabolism and Gene Expression. Trends Plant Sci..

[75] Shvedunova M., Akhtar A. (2022). Modulation of cellular processes by histone and non-histone protein acetylation. Nat. Rev. Mol. Cell Biol..

[76] Liu X., Wang C., Liu W., Li J., Li C., Kou X., Chen J., Zhao Y., Gao H., Wang H. (2016). Distinct features of H3K4me3 and H3K27me3 chromatin domains in pre-implantation embryos. Nature.

[77] Zheng H., Huang B., Zhang B., Xiang Y., Du Z., Xu Q., Li Y., Wang Q., Ma J., Peng X. (2016). Resetting Epigenetic Memory by Reprogramming of Histone Modifications in Mammals. Mol. Cell.

[78] Inoue A., Jiang L., Lu F., Zhang Y. (2017). Genomic imprinting of *Xist* by maternal H3K27me3. Genes Dev..

[79] Chen Z., Zhang Y. (2020). Maternal H3K27me3-dependent autosomal and X chromosome imprinting. Nat. Rev. Genet..

[80] Zenk F., Loeser E., Schiavo R., Kilpert F., Bogdanović O., Iovino N. (2017). Germ line–inherited H3K27me3 restricts enhancer function during maternal-to-zygotic transition. Science.

[81] Matoba S., Wang H., Jiang L., Lu F., Iwabuchi K.A., Wu X., Inoue K., Yang L., Press W., Lee J.T. (2018). Loss of H3K27me3 Imprinting in Somatic Cell Nuclear Transfer Embryos Disrupts Post-Implantation Development. Cell Stem Cell.

[82] Allshire R.C., Madhani H.D. (2018). Ten principles of heterochromatin formation and function. Nat. Rev. Mol. Cell Biol..

[83] Leung D., Du T., Wagner U., Xie W., Lee A.Y., Goyal P., Li Y., Szulwach K.E., Jin P., Lorincz M.C. (2014). Regulation of DNA methylation turnover at LTR retrotransposons and imprinted loci by the histone methyltransferase Setdb1. Proc. Natl. Acad. Sci. USA.

[84] Wang M., Chen Z., Zhang Y. (2022). CBP/p300 and HDAC activities regulate H3K27 acetylation dynamics and zygotic genome activation in mouse preimplantation embryos. EMBO J..

[85] Wu K., Fan D., Zhao H., Liu Z., Hou Z., Tao W., Yu G., Yuan S., Zhu X., Kang M. (2023). Dynamics of histone acetylation during human early embryogenesis. Cell Discov..

[86] Zhang M., Zhai Y., Zhang S., Dai X., Li Z. (2020). Roles of N6-Methyladenosine (m(6)A) in Stem Cell Fate Decisions and Early Embryonic Development in Mammals. Front. Cell Dev. Biol..

[87] Geula S., Moshitch-Moshkovitz S., Dominissini D., Mansour A.A., Kol N., Salmon-Divon M., Hershkovitz V., Peer E., Mor N., Manor Y.S. (2015). m6A mRNA methylation facilitates resolution of naïve pluripotency toward differentiation. Science.

[88] Collignon E., Cho B., Furlan G., Fothergill-Robinson J., Martin S.-B., McClymont S.A., Ross R.L., Limbach P.A., Ramalho-Santos M. (2023). m(6)A RNA methylation orchestrates transcriptional dormancy during paused pluripotency. Nat. Cell Biol..

[89] Yao H., Gao C.-C., Zhang D., Xu J., Song G., Fan X., Liang D.-B., Chen Y.-S., Li Q., Guo Y. (2023). scm(6)A-seq reveals single-cell landscapes of the dynamic m(6)A during oocyte maturation and early embryonic development. Nat. Commun..

[90] Zhang M., Zhai Y., An X., Li Q., Zhang D., Zhou Y., Zhang S., Dai X., Li Z. (2024). DNA methylation regulates RNA m(6)A modification through transcription factor SP1 during the development of porcine somatic cell nuclear transfer embryos. Cell Prolif..

[91] Zhang H., Wang Y., Hu Z., Wu Y., Chen N., Zhu Y., Yu Y., Fan H., Wang H. (2024). Zygotic Splicing Activation of the Transcriptome is a Crucial Aspect of Maternal-to-Zygotic Transition and Required for the Conversion from Totipotency to Pluripotency. Adv. Sci..

[92] Hocaoglu H., Wang L., Yang M., Yue S., Sieber M. (2021). Heritable shifts in redox metabolites during mitochondrial quiescence reprogramme progeny metabolism. Nat. Metab..

[93] Zhou L.T., Romar R., Pavone M.E., Soriano-Úbeda C., Zhang J., Slawson C., Duncan F.E. (2019). Disruption of *O*-GlcNAc homeostasis during mammalian oocyte meiotic maturation impacts fertilization. Mol. Reprod. Dev..

[94] Zhang L., Zhao J., Lam S.M., Chen L., Gao Y., Wang W., Xu Y., Tan T., Yu H., Zhang M. (2024). Low-input lipidomics reveals lipid metabolism remodelling during early mammalian embryo development. Nat. Cell Biol..

[95] Sudano M.J., Rascado T.D., Tata A., Belaz K.R., Santos V.G., Valente R.S., Mesquita F.S., Ferreira C.R., Araújo J.P., Eberlin M.N. (2016). Lipidome signatures in early bovine embryo development. Theriogenology.

[96] Gao M., Chen M., Chen Q., Zhu S., Wang H., Yang W., Wang X., Wang Q., Gu L. (2023). Integration of parallel metabolomics and transcriptomics reveals metabolic patterns in porcine oocytes during maturation. Front. Endocrinol..

[97] Birsoy K., Wang T., Chen W.W., Freinkman E., Abu-Remaileh M., Sabatini D.M. (2015). An Essential Role of the Mitochondrial Electron Transport Chain in Cell Proliferation Is to Enable Aspartate Synthesis. Cell.

[98] Zhu J., Schwörer S., Berisa M., Kyung Y.J., Ryu K.W., Yi J., Jiang X., Cross J.R., Thompson C.B. (2021). Mitochondrial NADP(H) generation is essential for proline biosynthesis. Science.

[99] Nikiforov A., Kulikova V., Ziegler M. (2015). The human NAD metabolome: Functions, metabolism and compartmentalization. Crit. Rev. Biochem. Mol. Biol..

[100] Biggers J.D., Whittingham D.G., Donahue R.P. (1967). The pattern of energy metabolism in the mouse oocyte and zygote. Proc. Nat. Acad. Sci. USA.

[101] Sharpley M.S., Chi F., Hoeve J.T., Banerjee U. (2021). Metabolic plasticity drives development during mammalian embryogenesis. Dev. Cell.

[102] Barbehenn E.K., Wales R.G., Lowry O.H. (1974). The Explanation for the Blockade of Glycolysis in Early Mouse Embryos. Proc. Natl. Acad. Sci. USA.

[103] Nagaraj R., Sharpley M.S., Chi F., Braas D., Zhou Y., Kim R., Clark A.T., Banerjee U. (2017). Nuclear Localization of Mitochondrial TCA Cycle Enzymes as a Critical Step in Mammalian Zygotic Genome Activation. Cell.

[104] Li J., Hou W., Zhao Q., Han W., Cui H., Xiao S., Zhu L., Qu J., Liu X., Cong W. (2024). Lactate regulates major zygotic genome activation by H3K18 lactylation in mammals. Natl. Sci. Rev..

[105] Merkuri F., Rothstein M., Simoes-Costa M. (2024). Histone lactylation couples cellular metabolism with developmental gene regulatory networks. Nat. Commun..

[106] Lu V., Roy I.J., Teitell M.A. (2021). Nutrients in the fate of pluripotent stem cells. Cell Metab..

[107] Gu W., Gaeta X., Sahakyan A., Chan A.B., Hong C.S., Kim R., Braas D., Plath K., Lowry W.E., Christofk H.R. (2016). Glycolytic Metabolism Plays a Functional Role in Regulating Human Pluripotent Stem Cell State. Cell Stem Cell.

[108] Nutt L.K., Margolis S.S., Jensen M., Herman C.E., Dunphy W.G., Rathmell J.C., Kornbluth S. (2005). Metabolic Regulation of Oocyte Cell Death through the CaMKII-Mediated Phosphorylation of Caspase-2. Cell.

[109] Meister A. (1983). Selective Modification of Glutathione Metabolism. Science.

[110] Bertoldo M.J., Listijono D.R., Ho W.-H.J., Riepsamen A.H., Goss D.M., Richani D., Jin X.L., Mahbub S., Campbell J.M., Habibalahi A. (2020). NAD+ Repletion Rescues Female Fertility during Reproductive Aging. Cell Rep..

[111] Chi F., Sharpley M.S., Nagaraj R., Roy S.S., Banerjee U. (2020). Glycolysis-Independent Glucose Metabolism Distinguishes TE from ICM Fate during Mammalian Embryogenesis. Dev. Cell.

[112] Sutton-McDowall M.L., Gilchrist R.B., Thompson J.G. (2004). Cumulus expansion and glucose utilisation by bovine cumulus–oocyte complexes during *in vitro* maturation: The influence of glucosamine and follicle-stimulating hormone. Reproduction.

[113] Frank L., Sutton-McDowall M., Brown H., Russell D., Gilchrist R., Thompson J. (2014). Hyperglycaemic conditions perturb mouse oocyte in vitro developmental competence via beta-O-linked glycosylation of Heat shock protein 90. Hum. Reprod..

[114] Guinez C., Morelle W., Michalski J.-C., Lefebvre T. (2005). O-GlcNAc glycosylation: A signal for the nuclear transport of cytosolic proteins?. Int. J. Biochem. Cell Biol..

[115] Zhang H., Qi J., Pei J., Zhang M., Shang Y., Li Z., Wang Y., Guo J., Sun K., Fan J. (2022). O-GlcNAc modification mediates aquaporin 3 to coordinate endometrial cell glycolysis and affects embryo implantation. J. Adv. Res..

[116] Zhong J., Li J., Burton G.J., Koistinen H., Cheung K.W., Ng E.H.Y., Yao Y., Yeung W.S.B., Lee C.-L., Chiu P.C.N. (2024). The functional roles of protein glycosylation in human maternal–fetal crosstalk. Hum. Reprod. Updat..

[117] Dalbies-Tran R., Cadoret V., Desmarchais A., Elis S., Maillard V., Monget P., Monniaux D., Reynaud K., Saint-Dizier M., Uzbekova S. (2020). A Comparative Analysis of Oocyte Development in Mammals. Cells.

[118] Paczkowski M., Schoolcraft W.B., Krisher R.L. (2014). Fatty acid metabolism during maturation affects glucose uptake and is essential to oocyte competence. Reproduction.

[119] Dunning K.R., Cashman K., Russell D.L., Thompson J.G., Norman R.J., Robker R.L. (2010). Beta-Oxidation Is Essential for Mouse Oocyte Developmental Competence and Early Embryo Development1. Biol. Reprod..

[120] Sanchez-Lazo L., Brisard D., Elis S., Maillard V., Uzbekov R., Labas V., Desmarchais A., Papillier P., Monget P., Uzbekova S. (2014). Fatty Acid Synthesis and Oxidation in Cumulus Cells Support Oocyte Maturation in Bovine. Mol. Endocrinol..

[121] Li L., Zhu S., Shu W., Guo Y., Guan Y., Zeng J., Wang H., Han L., Zhang J., Liu X. (2020). Characterization of Metabolic Patterns in Mouse Oocytes during Meiotic Maturation. Mol. Cell.

[122] Calder M.D., Chen R., MacDonald A., MacNeily Z., Leung Z.C.L., Adus S., Cui S., Betts D.H., Abu Rafea B., Watson A.J. (2022). Effects of palmitic acid on localization of embryo cell fate and blastocyst formation gene products. Reproduction.

[123] Shahzad Q., Pu L., Wadood A.A., Waqas M., Xie L., Pareek C.S., Xu H., Liang X., Lu Y. (2020). Proteomics Analysis Reveals that Warburg Effect along with Modification in Lipid Metabolism Improves In Vitro Embryo Development under Low Oxygen. Int. J. Mol. Sci..

[124] Yi Y., Lan X., Li Y., Yan C., Lv J., Zhang T., Jiang W. (2023). Fatty acid synthesis and oxidation regulate human endoderm differentiation by mediating SMAD3 nuclear localization via acetylation. Dev. Cell.

[125] van der Weijden V.A., Stötzel M., Iyer D.P., Fauler B., Gralinska E., Shahraz M., Meierhofer D., Vingron M., Rulands S., Alexandrov T. (2024). FOXO1-mediated lipid metabolism maintains mammalian embryos in dormancy. Nat. Cell Biol..

[126] Lipinska P., Pawlak P., Warzych E. (2023). Species and embryo genome origin affect lipid droplets in preimplantation embryos. Front. Cell Dev. Biol..

[127] Aizawa R., Ibayashi M., Mitsui J., Tsukamoto S. (2024). Lipid droplet formation is spatiotemporally regulated in oocytes during follicular development in mice. J. Reprod. Dev..

[128] Aizawa R., Ibayashi M., Tatsumi T., Yamamoto A., Kokubo T., Miyasaka N., Sato K., Ikeda S., Minami N., Tsukamoto S. (2019). Synthesis and maintenance of lipid droplets are essential for mouse preimplantation embryonic development. Development.

[129] Mau K.H.T., Karimlou D., Barneda D., Brochard V., Royer C., Leeke B., de Souza R.A., Pailles M., Percharde M., Srinivas S. (2022). Dynamic enlargement and mobilization of lipid droplets in pluripotent cells coordinate morphogenesis during mouse peri-implantation development. Nat. Commun..

[130] Wu Y., Chen K., Li L., Hao Z., Wang T., Liu Y., Xing G., Liu Z., Li H., Yuan H. (2022). Plin2-mediated lipid droplet mobilization accelerates exit from pluripotency by lipidomic remodeling and histone acetylation. Cell Death Differ..

[131] Arena R., Bisogno S., Gąsior Ł., Rudnicka J., Bernhardt L., Haaf T., Zacchini F., Bochenek M., Fic K., Bik E. (2021). Lipid droplets in mammalian eggs are utilized during embryonic diapause. Proc. Natl. Acad. Sci..

[132] Lipowsky R. (2014). Remodeling of membrane compartments: Some consequences of membrane fluidity. Biol. Chem..

[133] Wiktorowska-Owczarek A., Berezińska M., Nowak J.Z. (2015). PUFAs: Structures, Metabolism and Functions. Adv. Clin. Exp. Med..

[134] Yang T., Zhao J., Liu F., Li Y. (2022). Lipid metabolism and endometrial receptivity. Hum. Reprod. Update.

[135] Todorova P.K., Jackson B.T., Garg V., Paras K.I., Brunner J.S., Bridgeman A.E., Chen Y., Baksh S.C., Yan J., Hadjantonakis A.-K. (2024). Amino acid intake strategies define pluripotent cell states. Nat. Metab..

[136] Morris S.M. (2004). Enzymes of Arginine Metabolism. J. Nutr..

[137] Zhang Y., Bai J., Cui Z., Li Y., Gao Q., Miao Y., Xiong B. (2023). Polyamine metabolite spermidine rejuvenates oocyte quality by enhancing mitophagy during female reproductive aging. Nat. Aging.

[138] Lu V., Roy I.J., Torres A., Joly J.H., Ahsan F.M., Graham N.A., Teitell M.A. (2022). Glutamine-dependent signaling controls pluripotent stem cell fate. Dev. Cell.

[139] Betto R.M., Diamante L., Perrera V., Audano M., Rapelli S., Lauria A., Incarnato D., Arboit M., Pedretti S., Rigoni G. (2021). Metabolic control of DNA methylation in naive pluripotent cells. Nat. Genet..

[140] Dalton C.M., Szabadkai G., Carroll J. (2014). Measurement of ATP in Single Oocytes: Impact of Maturation and Cumulus Cells on Levels and Consumption. J. Cell. Physiol..

[141] Chen Q., Gao L., Li J., Yuan Y., Wang R., Tian Y., Lei A. (2022). *α*-Ketoglutarate Improves Meiotic Maturation of Porcine Oocytes and Promotes the Development of PA Embryos, Potentially by Reducing Oxidative Stress through the Nrf2 Pathway. Oxidative Med. Cell. Longev..

[142] Zhao J., Yao K., Yu H., Zhang L., Xu Y., Chen L., Sun Z., Zhu Y., Zhang C., Qian Y. (2021). Metabolic remodelling during early mouse embryo development. Nat. Metab..

[143] Jansen R.P. (2000). Origin and persistence of the mitochondrial genome. Hum. Reprod..

[144] Roth Z. (2018). Symposium review: Reduction in oocyte developmental competence by stress is associated with alterations in mitochondrial function. J. Dairy Sci..

[145] Sieber M.H., Thomsen M.B., Spradling A.C. (2016). Electron Transport Chain Remodeling by GSK3 during Oogenesis Connects Nutrient State to Reproduction. Cell.

[146] Rodríguez-Nuevo A., Torres-Sanchez A., Duran J.M., De Guirior C., Martínez-Zamora M.A., Böke E. (2022). Oocytes maintain ROS-free mitochondrial metabolism by suppressing complex I. Nature.

[147] Sullivan L.B., Gui D.Y., Hosios A.M., Bush L.N., Freinkman E., Vander Heiden M.G. (2015). Supporting Aspartate Biosynthesis Is an Essential Function of Respiration in Proliferating Cells. Cell.

[148] Miao Y., Cui Z., Gao Q., Rui R., Xiong B. (2020). Nicotinamide Mononucleotide Supplementation Reverses the Declining Quality of Maternally Aged Oocytes. Cell Rep..

[149] Nagano M., Katagiri S., Takahashi Y. (2006). ATP content and maturational/developmental ability of bovine oocytes with various cytoplasmic morphologies. Zygote.

[150] Iwata H., Goto H., Tanaka H., Sakaguchi Y., Kimura K., Kuwayama T., Monji Y. (2011). Effect of maternal age on mitochondrial DNA copy number, ATP content and IVF outcome of bovine oocytes. Reprod. Fertil. Dev..

[151] Hussein A.M., Wang Y., Mathieu J., Margaretha L., Song C., Jones D.C., Cavanaugh C., Miklas J.W., Mahen E., Showalter M.R. (2020). Metabolic Control over mTOR-Dependent Diapause-like State. Dev. Cell.

[152] DiTroia S.P., Percharde M., Guerquin M.-J., Wall E., Collignon E., Ebata K.T., Mesh K., Mahesula S., Agathocleous M., Laird D.J. (2019). Maternal vitamin C regulates reprogramming of DNA methylation and germline development. Nature.

[153] Chen B., Du Y.-R., Zhu H., Sun M.-L., Wang C., Cheng Y., Pang H., Ding G., Gao J., Tan Y. (2022). Maternal inheritance of glucose intolerance via oocyte TET3 insufficiency. Nature.

[154] Sutendra G., Kinnaird A., Dromparis P., Paulin R., Stenson T.H., Haromy A., Hashimoto K., Zhang N., Flaim E., Michelakis E.D. (2014). A Nuclear Pyruvate Dehydrogenase Complex Is Important for the Generation of Acetyl-CoA and Histone Acetylation. Cell.

[155] Liu W.-X., Liu H.-N., Weng Z.-P., Geng Q., Zhang Y., Li Y.-F., Shen W., Zhou Y., Zhang T. (2023). Maternal vitamin B1 is a determinant for the fate of primordial follicle formation in offspring. Nat. Commun..

[156] Lane A.N., Fan T.W.-M. (2015). Regulation of mammalian nucleotide metabolism and biosynthesis. Nucleic Acids Res..

[157] Leese H.J., Baumann C.G., Brison D.R., McEvoy T.G., Sturmey R.G. (2008). Metabolism of the viable mammalian embryo: Quietness revisited. Mol. Hum. Reprod..

[158] Fiscus J., Fraison É., Renault L., Salle B., Panthu B., Labrune E. (2024). Metabolic signature of follicular fluid in infertility-related diseases: A narrative review. Reprod. Biomed. Online.

[159] Kobayashi H., Imanaka S. (2024). Recent progress in metabolomics for analyzing common infertility conditions that affect ovarian function. Reprod. Med. Biol..

[160] Huang X., Dai J., Zhang H., Chen H., Han T.-L., Zou H., Zhang S. (2026). Integrated multi-omics analysis reveals epithelial-derived metabolic and adhesive dysregulation in the endometrium of patients with recurrent implantation failure. Eur. J. Med. Res..

[161] Boguenet M., Michel M., Bocca C., de la Barca J.M.C., Reynier P., May-Panloup P., Bouet P.-E. (2026). Endometrial metabolomic profiling reveals disruption of fatty-acid metabolism in unexplained recurrent pregnancy loss. Reprod. Biomed. Online.

[162] Yang X., Wang L., Wang R., Ding N., He Y., Wang F. (2025). Homocysteine induces endometrial ferroptosis via MAPK pathway in recurrent pregnancy loss. Placenta.

